# Impact of pharmacist-supported transition of care services in the Middle East and North Africa: a systematic review and meta-analysis

**DOI:** 10.1080/20523211.2024.2323099

**Published:** 2024-03-11

**Authors:** Eman N. Alhmoud, Safa Farooq Fouad Alrawi, Rasha El-Enany, Mohamed Izham Mohamed Ibrahim, Muhammad Abdul Hadi

**Affiliations:** a Pharmacy Department, Hamad Medical Corporation, Doha, Qatar; b Department of Clinical Pharmacy and Practice, College of Pharmacy, QU Health, Qatar University, Doha, Qatar

**Keywords:** Transitional care, pharmaceutical services, MENA region, healthcare utilization

## Abstract

**Background:**

Transition of care (TOC) is associated with an increased risk of medication-related problems. Despite recent advancements in pharmacy practice and research in the Middle East and North Africa (MENA), the characteristics and impact of regional pharmacy-supported TOC interventions remain unclear.

This systematic review and meta-analysis aimed to describe pharmacist-supported TOC interventions in the MENA region and evaluate their effectiveness.

**Methods:**

PubMed, CINAHL, EMBASE, Web of Science, World Health Organization’s International Clinical Trials Registry Platform (ICTRP) were searched from their inception to March 9, 2023, for experimental studies published in English, comparing pharmacist-supported TOC interventions with usual care for adults (age ≥18 years) discharged from the hospital. The risk of bias was evaluated using Cochrane’s risk-of-bias tool for randomised trials (ROB2) and the risk of bias in non-randomised studies of interventions (ROBINS-I) tool for randomised and non-randomised studies respectively. Narrative syntheses and meta-analysis methods were employed depending on the outcomes evaluated.

**Results:**

Twelve studies (n = 2377 subjects), 10 randomised controlled trials and 2 quasi-experimental studies, were included. Most studies had high or serious risk of bias. The included studies were quite heterogeneous in terms of nature and the delivery of intervention, and assessment of outcome measures. Compared to the usual care group, pharmacist-led TOC interventions contributed to a significant reduction in preventable drug-related (N = 2) and cardiac-related healthcare utilisation (N = 1), a significant reduction in preventable adverse drug events (ADEs) (Odds ratio (OR) 0.34, 95% CI: 0.13-0.94) and an improvement in medication adherence. However, all-cause hospitalisation and medication discrepancies were not significantly reduced.

**Conclusion:**

Pharmacy-supported TOC interventions may improve patient outcomes in the MENA region. However, considering the limited quality of evidence and the variability in intervention delivery, future well-designed clinical trials are needed.

## Introduction

Transition of care (TOC), defined as a patient’s movement from one healthcare provider or setting to another, is associated with an increased risk of medication discrepancies, medication errors, and adverse drug events (ADEs) (Alqenae et al., 2020; Lehnbom et al., 2014; World Health Organization, 2019). It is estimated that up to one out of two adults discharged from the hospital to the community experience at least one medication error or unintentional medication discrepancy, and one out of five suffer an ADE (Alqenae et al., 2020).

Evidence from the Middle East indicates that up to one in every four discharged patients experiences at least one medication discrepancy (Alanazi et al., 2022). Furthermore, up to 24% of patients experience an ADE within two weeks of hospital discharge, 61% of which are preventable (Al-Ghamdi et al., 2012). There is growing evidence supporting the impact of interventions provided by pharmacists during care transitions on enhancing medication adherence, detecting and resolving medication-related problems, and reducing healthcare use (Harris et al., 2022; King et al., 2021; Stroud et al., 2019). A recent systematic review of US-based studies demonstrated up to 44.5% reduction in 30-day hospital readmission rates with pharmacist-led TOC programmes (Harris et al., 2022).

Considerably, the prevailing evidence predominantly originated from the developed Western world, primarily mirrors the healthcare systems of the studies encompassed and may not directly translate to other regions or transitional care (TOC) programmes. The MENA region is diverse in its healthcare systems’ structures, financing, and challenges (Katoue et al., 2022), as well as in the education, training, privileges, and practicesof pharmacists (Sallom et al., 2023). Despite advancements in clinical pharmacy education, practice, and research in the MENA region (Badreldin et al., 2020; Boura et al., 2022; Hammad et al., 2017; Obaid et al., 2022), there is a clear gap in understanding how pharmacists can optimise transitional care process. Developing an understanding of factors that can potentially influence transition of care process is critically important in order to offer tailored recommendations for improving transitional care practices in the region. Therefore, the aim of this systematic review and meta-analysis (SRMA) was to identify, critically appraise, and synthesise research evidence on the impact of pharmacist-supported TOC interventions in the MENA region compared with usual (standard) care.

## Materials and methods

This SRMA was conducted following an a-priori protocol registered under the International Prospective Register of Systematic Reviews [CRD42023425085] (Eman Alhmoud & Safa Alrawi) and the Preferred Reporting Items for Systematic Reviews and Meta-Analyses (PRISMA) guidelines (Page et al., 2021).

### Study selection

Eligible studies included adults (≥ 18 years old) discharged from the hospital (inpatient-stay or emergency visit) to a home or another healthcare facility in the MENA region⁣. Countries were selected based on the World Bank definition (The World Bank).

The intervention consisted of pharmacy-based interventions in TOC, performed solely by or in coordination with pharmacy personnel (pharmacists, pharmacy students, pharmacy technicians, pharmacy interns) over the TOC continuum (i.e. at admission, during stay, at discharge, and post-discharge). The comparator was usual or standard care, as defined in each study. The primary outcome was healthcare utilisation. Secondary outcomes included medication discrepancies, medication errors, preventable ADEs, and patient adherence to medications. The supplementary data file lists the elaborated definitions of the primary and secondary outcomes. Eligible study designs were RCTs, quasi-experimental studies with a control group, and controlled before-and-after studies. Only full-text articles were included. We excluded case studies, qualitative studies, and non-research articles (e.g. editorials, opinion papers).

### Search strategy

We searched PubMed, Embase biomedical research (Elsevier), Cumulated Index to Nursing and Allied Health Literature (CINAHL) Ultimate (EBSCOhost), Web of Science (Clarivate), and World Health Organization International Clinical Trials Registry Platform (ICTRP) from the inception to the date of search (March09, 2023), using a combination of database-controlled vocabulary where available and free text keywords. We also searched for grey literature using ProQuest Dissertations, Thesis Global and Google Scholar.

No filters or restrictions on the language of publication were applied. The reference lists of articles retrieved for full review were also searched to identify any additional studies.

The complete search strategy for each database is listed in the supplementary data file.

### Selection of studies

Search results from databases and registers were combined and updated on EndNote (20) and then exported to Rayyan (www.rayyan.ai) where duplicate records were removed. Title and abstract screening were followed by full-article screening, performed by two reviewers (EA, SA) independently. Disagreements between reviewers were resolved through discussions.  

### Data extraction and management

The studies that fulfilled the inclusion criteria, two reviewers (EA, SA) independently extracted relevant data into specifically designed forms in Microsoft Excel and Microsoft Word. Each reviewer's extracted data were double-checked by another reviewer for accuracy. Disagreements were resolved through discussion and consensus. The extracted data included study characteristics, study design, participant characteristics, description of the intervention and the usual care groups, and outcomes.

We emailed the primary investigators of eight studies to obtain information about the study design, methods, and missing data. Additional data were successfully obtained from two investigators.

### Risk of bias (ROB) assessment

Two reviewers (EA, SA) independently assessed ROB in the included studies using the Revised Cochrane risk-of-bias tool for randomised trials (RoB 2) and the Risk Of Bias In Non-randomised Studies of Interventions (ROBINS-I) tools for RCTs and nonrandomized trials, respectively (Sterne et al., 2016; Sterne et al., 2019). The RoB2 tool is domain-based and consists of five domains. For Domain 2, we focused on evaluating the effect of assignment to the interventions at baseline.  Based on the domains’ ratings, the risk of bias in RCTs was rated as low, high, or some concerns. The ROBINS-I tool covers seven domains that address issues arising pre- and post-intervention. Based on the domains’ ratings, the risk of bias was rated as low, moderate, serious, critical, or no information. For non-randomised studies, potential confounders (demographics, comorbidities, prior healthcare utilisation, complexity of medication regimens) and co-interventions (receipt of additional care by other healthcare professionals) were set a priori. Reasons supporting the reviewers’ ROB judgment were reported. Disagreements were resolved by discussion and consensus among the reviewers.

### Data synthesis

Continuous outcomes were presented as means with their corresponding standard deviations (SD) or medians and interquartile ranges (IQR). Dichotomous outcomes were calculated as the number of participants with at least one event for each group and the corresponding percentages.

The heterogeneity of study populations, interventions, and outcome measurement precluded pooling of data by meta-analysis for most studies included in this systematic review. A meta-analysis was conducted for two consistently reported predefined outcomes. Effect estimates were calculated as odds ratios (OR) with corresponding 95% confidence interval (CI). Treatment effect estimates were pooled using a random-effects model to account for between-study variability and presented in forest plots. Between-study variation (Tau2) was estimated using the restricted maximum likelihood approach (REML). For pooled results, heterogeneity was assessed using the standard χ² test and I^2^ statistic (Deeks et al., 2019). We also attempted to investigate heterogeneity informally by ordering tables by hypothesised modifiers (study design and intervention characteristics). We did not perform subgroup analysis or sensitivity analysis because of the small number of studies included in the meta-analyses.

A *P*-value < 0.05 was considered statistically significant. All statistical analyses were conducted using Stata version 17. (StataCorp. 2021. Stata Statistical Software, Release 17. College Station, TX: StataCorp LLC).

We contacted the principal investigators of three studies (Al-Hashar et al., 2017; El Hajj et al., 2023; Salameh et al., 2019) to request missing data but failed to obtain any additional data.

Key outcomes from each study are summarised and presented in a tabular format.

The small number of studies included in the meta-analyses precluded formal assessment of publication bias (Page et al., 2019).

## Results

### Results of the search

**Figure 1.**
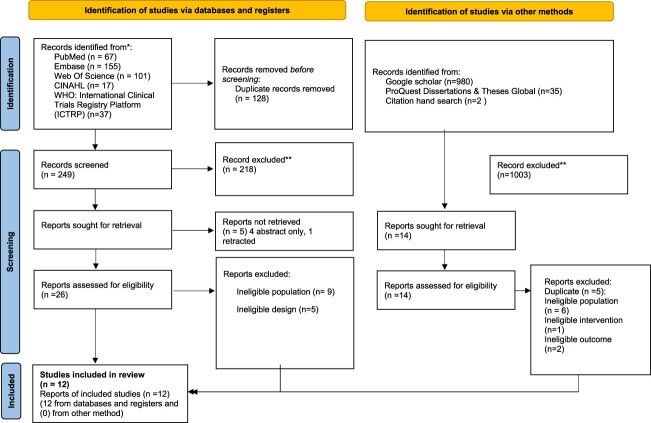
PRISMA flow diagram.

A PRISMA flowchart of the study inclusion is displayed in Figure 1.

The initial database/registers search identified 377 records. Of the 26 articles retrieved for full-text evaluation, 14 did not meet the inclusion criteria (ineligible population (n = 9), ineligible study design (n = 5)) and 12 were included. A grey literature search yielded 1017 records, of which 14 were retrieved for full-text evaluation and none were included in the review.

### Risk of bias

**Figure 2.**
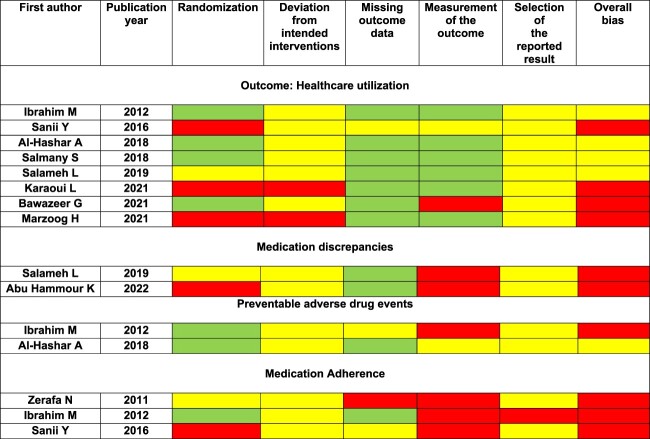
Risk of bias in RCTs.

The ROB for RCTs (N = 10) was judged as high for 10 of 15 outcomes. The remaining five outcomes had ‘some concerns’ regarding bias (Figure 2).

**Figure 3.**
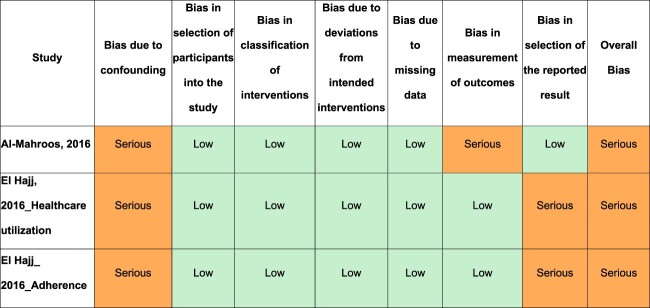
Assessing risk of bias in quasi-experimental studies.

For quasi-experimental studies, ROB was ‘serious’ for the three outcomes evaluated in two studies. This was mainly due to the failure to control for potential confounders (Figure 3).

Detailed justification for each ROB judgment is provided in the supplementary data file.

### Study characteristics

A summary of study characteristics, populations, and outcomes is provided in Table 1, grouped according to the location of intervention delivery and year of publication.

**Table 1.** Characteristics and outcomes of studies (arranged by type of interventions and publication year).

Author, publication Year, Country, registration number (if available)	Duration, Follow up	(Sample Size)	Patient Characteristics/		Targeted Recruitment (Inclusion criteria)	Outcomes	Overall effect	Risk of bias assessment
			Age (years) mean (SD*)	Gender (male) %				
**Randomised Controlled trials (RCTs)**								
**Interventions offered during hospital admission**								
Zerafa N (Zerafa et al., 2011) 2011 Malta	August 2008 – February 2009, 8-weeks	N = 80 I:40 C:40	I: 60.8 (7.8) C: 63.4 (8.7)	I: 72.5% C: 82.5%	Age >18 years, who underwent coronary artery bypass or heart valve surgery, discharged from cardiac surgical ward	**Adherence (compliance) to medications**: Mean percentage compliance to the knowledge of dose, dosage interval and instructions: I: 88.2% (95%CI 83.3 - 93.2) C: 66.4% (95%CI 59.0-73.9) *P* < 0.001 Compliance with regards to missing doses (number(n) (percentage)) of patients: I: 35 (87.5%) vs. C: 27 (67.5%), *P* = 0.032 Compliance to taking medicines at prescribed times (n (percentage)) of patients: I: 20 (50%) vs. C:12 (30%), *P* = 0.009 Compliance to stopping medication abruptly (n (percentage)) of patients: I: 36 (90%) vs. C: 31 (77.5%), *P* = 0.146	Favors intervention Favors intervention Favors intervention Neutral	High
Al-Hashar A (Al-Hashar et al., 2018) 2018 Oman **NCT02805270**	End of January 2014 – end of January 2015, 30-days	N = 587 I = 286 C: 301	I: 56 (17) C:57 (17)	I: 43% C: 42%	Admitted to medical wards for at least 24 h, ≥ one medication prior to admission, they or their caregiver spoke Arabic or English, could be interviewed for medication history	**Preventable adverse drug events (ADE) (primary outcome):** Total number of ADE (number/patient): I: 27 (0.09/patient) C: 59 (0.20/patient) *P* = 0.008 Patients with at least one preventable ADE (n (percentage)): I: 26 (9.1%) vs. C: 49 (16%), *P* = 0.009 Hospitalisation due to preventable ADE (n (percentage)) of patients: I: 6 (2.1%) vs. C: 16 (5.3%), *P* = 0.040	Favors intervention Favors intervention Favors intervention	Some concerns
						**Healthcare utilisation (secondary outcome):** Number of ED** visits, median (IQR): I: 46, 0 (0, 0) vs. C: 59, 0 (0, 0), *P* = 0.344 Number of unplanned hospital visits, median (IQR): I: 50, 0 (0, 0) vs. C: 61, 0 (0, 0), *P* = 0.639 Number of unplanned hospital readmissions, median (IQR): I: 39, 0 (0, 0) vs. C: 44, 0 (0, 0), *P* = 0.907 Number of ED visits, hospital readmission, and unplanned hospital visits, combined. median (IQR): I: 132, 0 (0, 1) vs. C: 165, 0 (0, 1), *P* = 0.193	Neutral	Some concerns
Salameh L (Salameh et al., 2019) 2019 Jordan	April – July 2017 Outcome assessed at hospital discharge, and at 30-days	N = 200 I:102 C: 98	I: 62.3 (15.6) C: 63.9 (13.4)	I: 56.9% C: 54.1%	Age ≥18 years, admitted in internal medicine department subdivisions, ≥ 4 regular pre-admission medications, expected hospital length of stay >48 h, spoke Arabic, no cognitive deficiency, not involved in any other clinical trial.	**Reduction in unintentional medication discrepancies/patient** (admission vs. discharge, expressed as mean (SD)): I: 0.82 (SD = 0.95) medication discrepancies/patient at admission vs. 0.58 (1.31) at discharge, *P* = 0.014 C: 0.61 (0.89) medication discrepancies/patient at admission vs. 0.82 (SD = 1.39) at discharge, *P* = 0.508 (Only within group, no between group comparison)	Favors intervention	High
						**Healthcare utilisation,** n (percentage) of patients: ED visits: I: 8 (7.8%) vs. C: 15 (15.3%), *P* = 0.098 Hospital readmission: I: 3 (2.9%) vs. C: 7 (7.1%), *P* = 0.207	Neutral	Some concerns
Abu Hammour K (Abu Hammour et al., 2022) 2022 Jordan **NCT 03928106**	July 2017 – July 2018 Outcome assessed at hospital discharge	N = 123 I: 61 C: 62	I: 62.1 (8.6) C: 61.8 (11.3)	I: 52.5% C: 50.0%	Admitted to surgical units, ≥ 4 regular long-term medications pre-admission, expected to stay in hospital for at least 48 h, spoke Arabic, no cognitive impairment	**Reduction in unintentional medication discrepancies/patient** (admission vs. discharge, mean (SD)): I: 0.86 (1.40) medication discrepancies/patient at admission vs. 0.68 (1.35) at discharge. Reduction (within group): 0.18 (0.43) *P* = 0.002 C: 0.53 (0.65) medication discrepancies/patient at admission vs. 0.41 (0.62) at discharge. Reduction (within group): 0.11 (0.32) *P* = 0.007 *P* value **comparing the 2 groups**: Admission (0.09), discharge (0.16), reduction (0.33)	Neutral	High
**Interventions commenced during hospital admission that include continuing support post-discharge**								
Ibrahim M (Ibrahim, 2012) 2012 Egypt	April 2009 – March 2010, 30-days (±2 days)	N = 250 I:125 C: 125	I: 62.7 (18.3) C: 59.8 (16.8)	I: 52.8% C: 55.2%	Admitted to general medicine service, discharged home, could be followed up by phone 30 days after discharge	**Preventable ADE (primary outcome):** Patients with at least one preventable ADE (n (percentage)): I: 4 (3%) vs. C: 18 (14%), *P* = <0.05	Favors intervention	High
						**Healthcare utilisation** (n (percentage)) of patients: ED visit or readmission: I: 30 (24%) vs. C: 35 (28%), *P* = NS (not reported) Medication related ED visit or readmission: I: 8 (6%) vs. C: 10 (8%), *P* = NS (not reported) Preventable medication-related ED visits or hospital readmissions: I: 3 (2%) vs. C: 11 (9%), *P* = 0.03	Neutral Neutral Favors intervention	Some concerns
						**Adherence to medications:** Non-adherent with at least 1 medication, (n (percentage)) of patients: I: 16 (13%) vs. C: 30 (24%), *P* = 0.026	Favors intervention	High
Sanii Y (Sanii et al., 2016) 2015 Islamic Republic of Iran (IRI)	August 2013 – March 2014, 1-month	N = 154 I: 78 C: 76	I: 52.3 (13.1) C: 54.8 (13.8)	I: 56.4% C: 60.5%	Age 18–65 years, discharged from respiratory ward on ≥ 3 medications, any target drugs (inhalers, anti- hypertensive, digoxin, or antiplatelets), minimum hospital stay of 2 days, patient, or caregiver able to write and read in Persian language	**Adherence to inhaler medication (primary outcome),** measured by Medication adherence rating scales (MARS) questionnaires (10 questions), mean (SD), (mean rank): I: 93.2 (9.2), (98.78) vs. C: 50.3 (27.1), (54.35), *P* = 0.010	Favors intervention	High
						**Healthcare utilisation:** Medication-related ED visits or hospital readmission (n (percentage)) of patients: I: 0 (0%) vs. C: 8 (10.5%), no *P* value.	NA	High
Salmany S (Salmany et al., 2018) 2017 Jordan	March – May 2015, 30-days	N = 332 I:166 C: 166	I: 47.2 (16) C: 49.2 (16)	I: 46.4% C:48.2%	Oncology patients who were discharged from inpatient services on weekdays (Sunday through Thursday)	**Healthcare utilisation,** (n (percentage)) of patients: ED visits: I: 63 (44%) vs. C: 80 (52%), *P* = 0.123 Hospital readmission: 57 (37%) vs. C: 66 (43%), *P* = 0.317	Neutral	Some concerns
Karaoui L (Karaoui et al., 2021) 2021 Lebanon **LBCTR2020033424**	August 2017 – July 2019, 30-days	N = 200 I:100 C:100	I: 74.7 (12.09) C: 73.2 (14.74)	I: 46% C: 43%	Age ≥18 years, discharged on oral anticoagulant for a therapeutic indication, able to communicate in Arabic or English	**Healthcare utilisation (primary outcome):** All-cause readmission rates including unplanned physician’s clinic visit, (n (percentage)) of patients: I: 15 (15%) vs. C: 12 (12%), OR¶: 0.847 (95% CI 0.380–1.886), *P* = 0.802 Readmission related to anticoagulant use (n (percentage)) of patients: I: 7% vs. C: 7%, OR 0.684 (95% CI 0.193–2.419), *P* = 0.650	Neutral	High
Marzoog H (Marzoog et al., 2021) 2021 Iraq	August 2018 – April 2019, 12-weeks	N = 50 I: 25 C: 25	I: 57 (14.6) C: 61.9 (9.6)	I: 56% C: 64%	Patients admitted with acute heart failure	**Healthcare utilisation:** hospital readmission for acute heart failure (n (percentage)) of patients I: 6 (24%) vs C: 15 (60%), *P* = Not calculated.	NA	High
Bawazeer G (Bawazeer et al., 2021) 2021 Kingdom of Saudi Arabia (KSA)	October 2016 – April 2017, 30-days	N = 98 I = 56 C: 51	I: 52.9 (15.9) C: 53.8 (15.41)	I: 41% C: 38%	Age ≥18 years, discharged on insulin, warfarin, or both, eligible for outpatient follow-up at the study site	**Healthcare utilisation (primary outcome):** hospital readmission rate (n (percentage)) of patients: I: 8 (15%) vs. C: 11 (23%), *P* = 0.48. Time to first unplanned healthcare use: HR 0.49 (95% CI, 0.19–1.24); *P* = 0.12	Neutral Neutral	High
**Quasi-experimental studies**								
**Interventions commenced during hospital admission that include continuing support post-discharge**								
El Hajj M (El Hajj et al., 2023) 2023 Qatar NCT02648243	March 2016 – December 2017, 6-months	N = 373 I: 111 Usual care: 120 C: 142	I: 51.5 (11.1) Usual care: 54.8 (11.5) C: 52.3 (10.2)	I: 95.5% Usual care: 83.3% C: 88%	Age ≥18 years, admitted to and discharged from non-surgical cardiology ward with a diagnosis of ACS, communicating in English and/or Arabic	**Healthcare utilisation (primary outcome): Cardiac related readmission:** (n (percentage)) of patients: I: 12 (10.8%), usual care: 43 (35.8%), C:42 (29.6%) Usual care vs. intervention (reference): adjusted OR 1.939, (95%CI 0.913-4.117), *P* = 0.085 Control vs. intervention (reference): adjusted OR 2.428 (95% CI 1.116-5.282), *P* = 0.025* Usual care + control vs. intervention (reference): adjusted OR 2.140 (95% CI 1.062-4.312), *P* = 0.033* All-cause hospitalisations (n (percentage)) of patients: I:19 (17.1%), usual care: 43 (35.8%), C: 42(29.6%) Usual care vs. intervention (reference): adjusted OR 1.701, (95%CI 0.888-3.257), *P* = 0.109 Control vs. intervention (reference): adjusted OR 1.744 (95% CI 0.876-3.474), *P* = 0.114 Usual care + control arm vs. intervention (reference): adjusted OR 1.719 (95% 0.941-3.138), *P* = 0.078	Neutral Favors intervention Favors intervention Neutral Neutral Neutral	Serious
						Adherence to evidence-based secondary prevention therapy (Proportion of days covered (PDC), prescription refill records at outpatient pharmacy), number of patients (percentage) with ADC > 75% I: 65 (60.7%) vs. Usual care: 72 (60.0%) vs. C: 68 (50.0%), *P* = 0.156 (unadjusted)	Neutral	Serious
**Interventions commenced post-discharge**								
Al-Mahroos M (Al-Mahroos et al., 2017) 2017 Iraq	Not listed, 4 visits: 8-10, 30, 60, and 90 – days	N = 50 I: 27 C:23	I: 46.9 (12.1) C: 52.8 (10.5)	I: 55% C: 39%	Patients discharged from hospital on warfarin for deep vein thrombosis, pulmonary embolism, atrial fibrillation. and valve replacement	Patient adherence to warfarin at 30–60 and 90-days: n (percentage) of patients: with good adherence: I:27 (100%)−27 (100%) – 27(100%) C: 20 (87.0%) – 20 (87.0%) – 19 (82.6%), respectively (unadjusted)	Favors intervention	Serious

*SD: standard deviation, **ED: emergency department, ¶ OR: Odds ratio

The twelve retrieved studies included 10 RCTs (Abu Hammour et al., 2022; Al-Hashar et al., 2018; Bawazeer et al., 2021; Ibrahim, 2012; Karaoui et al., 2021; Marzoog et al., 2021; Salameh et al., 2019; Salmany et al., 2018; Sanii et al., 2016; Zerafa et al., 2011),1 pilot RCT(Bawazeer et al., 2021), and 2 quasi-experimental studies (Al-Mahroos et al., 2017; El Hajj et al., 2023). Studies were published between September 1, 2011 (Zerafa et al., 2011) and February 16, 2023 (El Hajj et al., 2023). Studies originated from nine different countries: Egypt (n = 1) (Ibrahim, 2012), Iran (n = 1) (Sanii et al., 2016), Iraq (n = 2) (Al-Mahroos et al., 2017; Marzoog et al., 2021), Jordan (n = 3) (Abu Hammour et al., 2022; Salameh et al., 2019; Salmany et al., 2018), Kingdom of Saudi Arabia (n = 1)(Bawazeer et al., 2021), Lebanon (n = 1)(Karaoui et al., 2021), Malta (n = 1)(Zerafa et al., 2011), Oman (n = 1)(Al-Hashar et al., 2018), and Qatar (n = 1)(El Hajj et al., 2023). In total, 2377 patients were included, with individual study sample sizes ranging from 50 (Al-Mahroos et al., 2017; Marzoog et al., 2021) patients to 587 patients (Al-Hashar et al., 2018).

The mean age of the patients ranged from 46.9 years (Al-Mahroos et al., 2017) to 74.7 years (Karaoui et al., 2021), and the percentage of male patients ranged from 38% (Bawazeer et al., 2021) to 95.5% (El Hajj et al., 2023).

The patient population between the studies varied, including patients discharged from specific units within hospitals (e.g. surgical unit (n = 1) (Abu Hammour et al., 2022), medical units (n = 3) (Al-Hashar et al., 2018; Ibrahim, 2012; Salameh et al., 2019)); those receiving high-risk medications (Al-Mahroos et al., 2017; Bawazeer et al., 2021; Karaoui et al., 2021), and individuals with specific diagnoses [cardiology (n = 3)] (El Hajj et al., 2023; Marzoog et al., 2021; Zerafa et al., 2011), oncology (n = 1) (Salmany et al., 2018).

### Characteristics of the intervention

A description of the intervention settings and characteristics is provided in Table 1a in the Supplementary Data file.

All interventions were initiated in the inpatient settings except in one study (Al-Mahroos et al., 2017). Most of these were academic/teaching hospitals (n = 9). All interventions were delivered by pharmacists, except in two studies where undergraduate pharmacy students delivered intervention (Bawazeer et al., 2021; Zerafa et al., 2011). Most of the studies implemented multiple interventions. The number of interventions implemented in each study ranged between one and four, with a median number of 2 interventions. Interventions delivered included the provision of bedside medication delivery (n = 1) (Al-Hashar et al., 2018), review of discharge prescriptions (n = 2) (Karaoui et al., 2021) (El Hajj et al., 2023), medication reconciliation (n = 7) (Abu Hammour et al., 2022; Al-Hashar et al., 2018; Bawazeer et al., 2021; El Hajj et al., 2023; Ibrahim, 2012; Salameh et al., 2019; Sanii et al., 2016), patient-centered post-discharge follow-up (n = 8) and discharge counselling and education (n = 10) (Al-Hashar et al., 2018; Bawazeer et al., 2021; El Hajj et al., 2023; Ibrahim, 2012; Karaoui et al., 2021; Marzoog et al., 2021; Salameh et al., 2019; Salmany et al., 2018; Sanii et al., 2016; Zerafa et al., 2011). Patient-centered post-discharge follow-up was provided over the phone in 6 studies (Bawazeer et al., 2021; El Hajj et al., 2023; Ibrahim, 2012; Karaoui et al., 2021; Salmany et al., 2018; Sanii et al., 2016) and by face-to-face clinic visits in two studies (Al-Mahroos et al., 2017; Marzoog et al., 2021).

The definition of ‘**usual care’** varied among the studies included. Four studies evaluated the effectiveness of intensive, structured pharmacist interventions compared with pharmacist-delivered routine standard-of-care services, such as medication review (Al-Hashar et al., 2018) and discharge counselling (Bawazeer et al., 2021; Ibrahim, 2012; Salmany et al., 2018). El-Hajj et al. (El Hajj et al., 2023) evaluated three groups: (1) an intervention group (structured clinical pharmacist-delivered TOC intervention), (2) a usual care group (usual care at discharge by clinical pharmacists), and (3) a control group (discharge education by nurses and/or physicians). The absence of pharmacist interventions was explicitly mentioned in the remaining studies.

### Fidelity of the intervention implementation data: (Montgomery et al., 2013)

Few studies have provided a description of the implementation data.

Only two studies have reported the actual length of follow-up phone calls (intervention dosage) (Bawazeer et al., 2021; Salmany et al., 2018). Only a few studies have reported the provision of training to study personnel (Abu Hammour et al., 2022; Bawazeer et al., 2021; El Hajj et al., 2023; Karaoui et al., 2021; Salameh et al., 2019) and the use of standardised materials (i.e. educational leaflets) (Bawazeer et al., 2021; El Hajj et al., 2023; Karaoui et al., 2021) and phone call scripts (Bawazeer et al., 2021; Karaoui et al., 2021; Salmany et al., 2018) to ensure consistency of intervention delivery.

None of the studies reported the risk of contamination and/or uptake of intervention components outside the trial context. Details of the interventions delivered to the control group are scarcely described. None of the studies reported qualitative data evaluating the experiences of intervention implementers or recipients.

## Outcomes

A detailed description is provided in the supplementary data file.

### Primary outcome: healthcare utilisation

Healthcare utilisation was reported in eight RCTs (n = 1871) (Al-Hashar et al., 2018; Bawazeer et al., 2021; Ibrahim, 2012; Karaoui et al., 2021; Marzoog et al., 2021; Salameh et al., 2019; Salmany et al., 2018; Sanii et al., 2016) and one quasi-experimental study (n = 373) (El Hajj et al., 2023). It was assessed at 30 days post-discharge in most of the trials (N = 7), using medical file review (Ibrahim, 2012; Marzoog et al., 2021; Salmany et al., 2018), patient interviews (Al-Hashar et al., 2018; Karaoui et al., 2021; Salameh et al., 2019), or both (Bawazeer et al., 2021).

Studies reported different definitions, including all-cause hospital readmission (N = 5) (Bawazeer et al., 2021; El Hajj et al., 2023; Karaoui et al., 2021; Salameh et al., 2019; Salmany et al., 2018), emergency department (ED) visit (N = 2) (Al-Hashar et al., 2018; Salameh et al., 2019), combined ED visit or hospital readmission (Al-Hashar et al., 2018; Ibrahim, 2012), cardiac related (El Hajj et al., 2023; Marzoog et al., 2021), medication related (Ibrahim, 2012; Karaoui et al., 2021; Sanii et al., 2016), and preventable medication related (Al-Hashar et al., 2018; Ibrahim, 2012) hospitalisations or ED visits.

#### Evidence from RCTs

**Figure 4.**
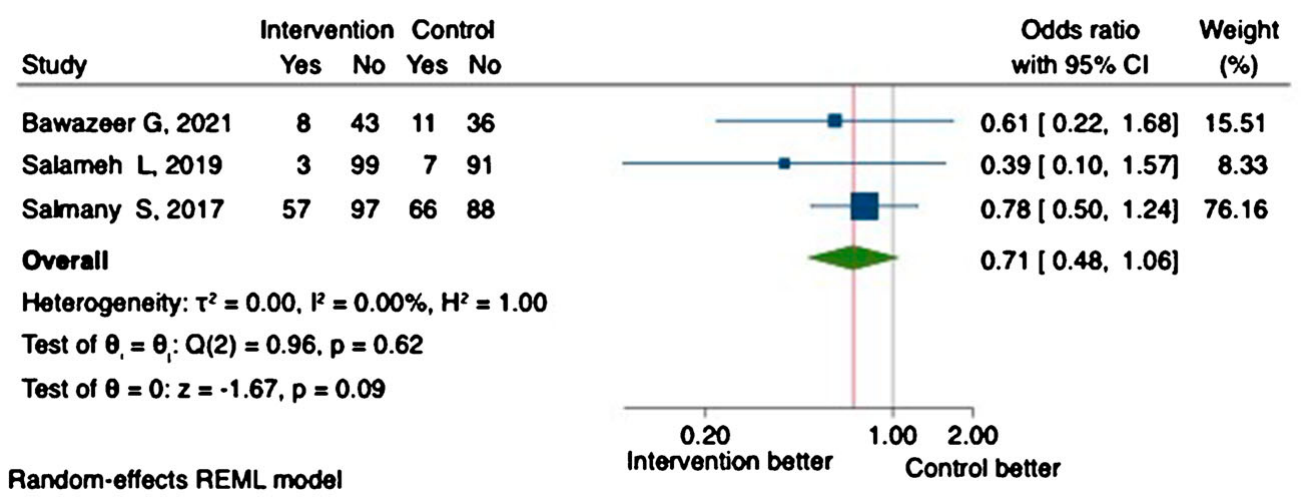
Proportion of patients with all-cause hospital readmission at 30-days.

Healthcare utilisation was the primary outcome in two RCTs (Bawazeer et al., 2021; Karaoui et al., 2021). Only two trials reported a significant reduction in preventable medication-related ED visits or hospitalisation (secondary outcomes) (Al-Hashar et al., 2018; Ibrahim, 2012). Notably, none of the trials was powered to detect a difference in healthcare utilisation. A meta-analysis of three RCTs demonstrated comparable all-cause hospital readmission at 30 days between the intervention and control arms (OR 0.71, 95% CI 0.48-1.06, *P* = 0.09) (Figure 4).

#### Evidence from quasi-experimental studies

Healthcare utilisation was the primary outcome in El Hajj et al. (El Hajj et al., 2023), which included 3 groups as described above. Patients in the control group experienced significantly higher odds of cardiac-related hospitalisation at 6 months (OR 2.428, 95% CI 1.116–5.282, *P* = 0.025) than those in the intervention group. All-cause hospitalisation, however, was comparable between the groups.

### Medication discrepancies

Two RCTs (n = 232) (Abu Hammour et al., 2022; Salameh et al., 2019) evaluated the impact of pharmacist-led medication reconciliation at admission on reducing unintentional medication discrepancies at discharge as a primary outcome. Both trials demonstrated a significant within-group reduction (change-from-baseline) in the mean number of unintentional medication discrepancies per patient with the intervention. Only one trial, however, reported the extent of the reduction through comparison between the intervention and control groups, which was not different (*P* = 0.33).

### Preventable ADEs:

**Figure 5.**
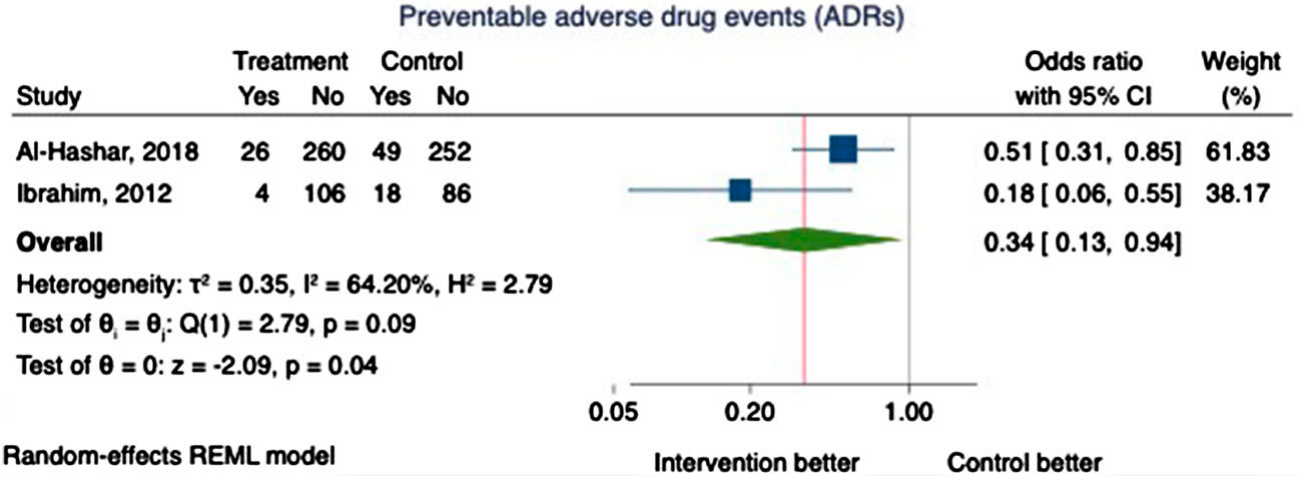
The proportion of patients experiencing at least one adverse drug event (ADE).

Two RCTs reported the incidence of preventable ADEs 30 days post-discharge as a primary outcome (n = 801) (Al-Hashar et al., 2018; Ibrahim, 2012). ADEs were identified through self-reporting by unblinded participants and medical chart review. Preventability was assessed by blinded clinical pharmacists who applied different criteria. Pooling of data demonstrated a 66% reduction in the odds of experiencing a preventable ADE in the intervention group (OR 0.34, 95% CI 0.13-0.94, *P* = 0.04), but this was associated with substantial heterogeneity (I^2 ^=  64.20%) (Figure 5).

### Adherence

Medication adherence was evaluated in three RCT (n = 484) (Ibrahim, 2012; Sanii et al., 2016; Zerafa et al., 2011) and 2 quasi-experimental studies (n = 423) (Al-Mahroos et al., 2017; El Hajj et al., 2023) at different time points (Table 1).

#### Evidence from RCTs

Medication adherence was the primary outcome of two RCTs (Sanii et al., 2016; Zerafa et al., 2011). It was measured using a researcher-developed questionnaire (Zerafa et al., 2011), self-reporting of unblinded participants (Ibrahim, 2012), and the medication adherence rating scales (MARS) questionnaire (Sanii et al., 2016).

The three trials reported significantly higher adherence in the intervention group. (Table 1).

Noteworthy, the version of the MARS questionnaire used to evaluate adherence to inhaler medications was not specified, and the reported result (mean (SD) adherence score) did not match that of the Medication Adherence Reporting Scale for Asthma (MARS-A), which defines high adherence as a score of 4.5 or higher (Horne & Hankins, 2004). This limits the interpretation of the current findings.

#### Evidence from quasi-experimental studies

In the study by Al-Hajj et al., adherence to acute coronary syndrome (ACS) secondary prevention medications, calculated using the proportion of days covered (PDC), was comparable between the three evaluated groups. On the other hand, Al Mahroos et al. (Al-Mahroos et al., 2017) found a significantly higher self-reported adherence to warfarin at 30,60 and 90 days in the intervention group compared with the control group. (Assessment method clarified by contacting the second author MKA).

## Discussion

In this systematic review, we examined the role of pharmacists in facilitating transition of care within the MENA region, critically appraised relevant literature and synthesised evidence in order to understand current practices and potential areas for improvement in transitional care process. The current review documented a wide spectrum of pharmacy-supported TOC interventions delivered across the continuum of care in the MENA region. The distribution of interventions described in this review aligns with those documented in a systematic review of US-based studies, with patient counselling, medication reconciliation, and patient-centered follow-up being the most prevalent interventions (Harris et al., 2022).

Healthcare utilisation was the most frequently reported outcome. This aligns with the increased recognition of its role as a quality indicator for care transitions and its impact on health system financing (James, 2013). Despite the significant reduction in preventable drug-related and cardiac-related healthcare utilisation, pharmacist-led TOC interventions did not reduce all -cause hospitalisations and/or ED visits, regardless of patient populations and practice settings. This contradicts the findings of a US-based systematic review by Harris et al., where pharmacy-led TOC programmes resulted in fewer hospital readmissions in 89.4% of the trials (Harris et al., 2022). Our findings may be attributed to the limited statistical power and reliance on pharmacy professionals as sole interveners. Ensing et al. demonstrated that collaborating with physicians and nurses enhanced the effectiveness of pharmacist-delivered TOC interventions, underscoring the complexity of patient care and the value of interdisciplinary collaboration (Ensing et al., 2015).

The lack of significant reduction in unintentional medication discrepancies with pharmacist lead-medication reconciliation compared with no intervention contrasts with a previously reported systematic review that showed a substantial reduction in medication discrepancies across Europe, Australia, and the Americas because of pharmacist interventions (Cheema et al., 2018). This could be due to the limited number of studies (1 RCT) and variations in physicians’ acceptance of pharmacists’ therapeutic recommendations to resolve identified medication discrepancies.

Another key finding of this SRMA is the significant reduction in preventable ADEs despite the use of different measurement methods and preventability criteria employed across the included studies. This finding aligns with results from an RCT conducted by Schnipper et al. (Schnipper et al., 2006), where pharmacist counselling and follow-up reduced the rate of preventable ADEs and medication-related readmissions but did not reduce all-cause healthcare utilisation. Identifying ADEs 30 days post-discharge raises the possibility of some relevant information not being recalled appropriately leading to under or over estimation.

Apart from the study by El Hajj et al. (El Hajj et al., 2023), the observed improvements in medication adherence with pharmacy-supported TOC interventions should be interpreted cautiously given the variability in the methods to assess medication adherence, especially the use of non-validated tools as these methods may introduce detection bias. Moreover, reliance on patient self-reporting has the potential to overestimate adherence rates secondary to social desirability bias. This highlights the need for future research to use standardised and validated methods to assess medication adherence.

This review is the first to present an evidence synthesis on pharmacist-led TOC interventions and assess their effectiveness in the MENA region. We applied a comprehensive search strategy and broad inclusion criteria that accounted for all interventions delivered by pharmacy professionals or students, regardless of patient characteristics or practice settings. However, there are a few limitations which should be carefully considered. First, there was significant heterogeneity in terms of research population, nature and delivery of intervention, outcome measurement, and follow-up duration, which limited our ability to combine results statistically through meta-analysis. Second, most of the studies were of low quality and suffered considerable methodological limitations leading to challenges in drawing definitive conclusions and generalising findings to broader healthcare contexts within the MENA region.

Moreover, the included studies lacked detailed descriptions of the usual care group, making it challenging to develop a consistent, precise definition of usual care in TOC research. Additionally, the scarcity of implementation fidelity data poses a challenge in determining the extent to which the interventions were implemented as intended and whether the lack of intervention benefit was attributable to the failure of the intervention or its implementation (Moore et al., 2015). In a systematic review of US-based pharmacy-led TOC interventions, more than three-quarters of the studies did not report intervention fidelity (Rodrigues et al., 2017). Additionally, readmissions may have been underestimated because of the restricted availability of readmission data from other hospitals and the fact that many of these studies were conducted at single hospitals. A limitation that was also raised by previous SRMA studies (Harris et al., 2022; Rodrigues et al., 2017).

### Implications for practice

While demonstrating the complexity and diversity of care transitions in the region, the current findings highlight the importance of considering pharmacy-supported interventions at TOC, their feasibility, and overall acceptability. However, given the heterogenity among the included studies and diversity in the nature and delivery of intervention, we could not determine which pharmacist intervention was the most useful. To achieve positive outcomes, pharmacist interventions may need to be tailored to and targeted at high-risk populations.

### Implications for future research

More well-designed and powered studies are needed to investigate the impact of pharmacist-led TOC interventions on healthcare utilisation, patient-centered and economic outcomes.

Studies reporting process evaluations of complex interventions are needed to provide a better understanding of the intervention implementation (fidelity, dose, reach), mechanism of action, and contextual factors that may affect the intervention implementation and outcomes. (Moore et al., 2015). Furthermore, evaluating the integration of comprehensive medication management within TOC warrants further investigation.

Additionally, identified research gaps encompass evaluating the impact of TOC interventions for patients discharged from the ED, which bridges inpatient and outpatient care, and investigating the perspectives and experiences of those implementing or receiving these interventions, along with their perceived facilitators and barriers, using mixed-methods approaches.

## Conclusion

This systematic review found that pharmacist-led interventions were effective in reducing preventable ADE-related and cardiac-related healthcare utilisation, preventable ADEs and improving medication adherence. However, these interventions did not result in significant reductions in other types of healthcare use or medication discrepancies. Because the quality of the studies included in this review was low, the findings should be interpreted with caution. The literature gaps identified warrant further research.

## Supplementary Material

Supplemental Material
