# Supplementary material for: Impact of pharmacist-supported transition of care services in the Middle East and North Africa: a systematic review and meta-analysis
Source: J Pharm Policy Pract. 2024 Mar 11;17(1):2323099. doi: 10.1080/20523211.2024.2323099 (PMC10930094; doi:10.1080/20523211.2024.2323099)
Supplement: Supplemental Material [file JPPP_A_2323099_SM6215.pdf]

**Supplementary data**

## Elaborated definitions for the outcomes:

| Outcome | Definition |
|---|---|
| Healthcare utilization | The use of healthcare services for preventing and curing health problems, promoting maintenance of health and well-being, or obtaining information about health status and prognosis (Orbell et al., 2013). |
| Medication discrepancies | Unclear changes that have been documented in the medication lists of patients during their movement across different sites of care (Coleman et al., 2002). |
| Medication errors | any preventable event that may cause or lead to inappropriate medication use or patient harm while the medication is in the control of the health care professional, patient, or consumer (National Coordinating Council for Medication Error Reporting and Prevention, 2023). |
| preventable ADEs | adverse events caused by an error or other type of systems or equipment failure (Leape et al., 1993). |
| Adherence to medications | the extent to which the patient's behavior (medication usage) matches agreed recommendations from the clinician (R. Horne, 2006). |

Search Strategy:

**Pubmed:**

| Search | Actions | Details | Query | Results |
|---|---|---|---|---|
| #31 | | | Search: **#7 AND #14 AND #19 AND #24 AND #30** Sort by: **Most Recent** | 67 |
| #30 | | | Search: **#28 OR #29** Sort by: **Most Recent** | 455,644 |
| #29 | | | Search: **'medication compliance'[Title/Abstract] OR compliance[Title/Abstract] OR adherence[Title/Abstract] OR "medication* error*"[Title/Abstract] OR "medication discrepanc*"[Title/Abstract] OR discrepanc*[Title/Abstract] OR"readmission*"[Title/Abstract] OR "re-admission*"[Title/Abstract] OR rehospitalization*[Title/Abstract] OR readmit*[Title/Abstract] OR 'hospital reutilization'[Title/Abstract]** Sort by: **Most Recent** | 430,317 |
| #28 | | | Search: **#25 OR #26 OR #27** Sort by: **Most Recent** | 66,959 |
| #27 | | | Search: **Medication Adherence[MeSH Terms]** Sort by: **Most Recent** | 25,383 |
| #26 | | | Search: **Medication Errors[MeSH Terms]** Sort by: **Most Recent** | 19,890 |
| #25 | | | Search: **Patient Readmission[MeSH Terms]** Sort by: **Most Recent** | 22,374 |
| #24 | | | Search: **#22 OR #23** Sort by: **Most Recent** | 1,176,883 |
| #23 | | | Search: **bahrain* OR iraq* OR kuwait* OR saudi* OR 'saudi arabia' OR oman* OR jordan* OR lebanese OR lebanon OR iran* OR 'gaza strip' OR palestin* OR 'west bank' OR syria* OR 'syrian arab republic' OR egypt* OR yemen* OR 'united arab emirates' OR emirat* OR 'eastern mediterranean region' OR 'middle east' OR 'middle-east' OR 'north africa' OR 'north-africa' OR mena OR qatar* OR algeria* OR tunisia* OR morroco* OR libya* OR djibouti* OR israel* OR malta*** Sort by: **Most Recent** | 1,176,883 |
| #22 | | | Search: **#20 OR #21** Sort by: **Most Recent** | 197,242 |
| #21 | | | Search: **Africa, Northern[MeSH Terms]** Sort by: **Most Recent** | 41,081 |
| #20 | | | Search: **Middle East[MeSH Terms]** Sort by: **Most Recent** | 159,292 |
| #19 | | | Search: **#17 OR #18** Sort by: **Most Recent** | 337,041 |
| #18 | | | Search: **"transition* of care"[Title/Abstract] OR 'transitional care'[Title/Abstract] OR 'hospital to home'[Title/Abstract] OR discharge*[Title/Abstract] OR "care transition*"[Title/Abstract]** Sort by: **Most Recent**<br>"transition of care"[Title/Abstract] OR "transitional care"[Title/Abstract] OR "hospital to home"[Title/Abstract] OR "discharge*"[Title/Abstract] OR "care transition*"[Title/Abstract] | 328,951 |
| #17 | | | Search: **#15 OR #16** Sort by: **Most Recent** | 39,464 |
| #16 | | | Search: **Patient Discharge[MeSH Terms]** Sort by: **Most Recent** | 38,620 |
| #15 | | | Search: **Transitional Care[MeSH Terms]** Sort by: **Most Recent** | 1,211 |

| | | | | |
|---|---|---|---|---|
| #14 | | | Search: **#12 OR #13** Sort by: **Most Recent** | 3,804,792 |
| #13 | | | Search: **Education[Title/Abstract] OR Counseling[Title/Abstract] OR Review[Title/Abstract] OR reconcile*[Title/Abstract] OR reconciliation[Title/Abstract] OR 'therapy management'[Title/Abstract] OR 'medication therapy management'[Title/Abstract] OR ‘Follow up’[Title/Abstract] OR ‘Follow-up’[Title/Abstract]** Sort by: **Most Recent**<br>"Education"[Title/Abstract] OR "Counseling"[Title/Abstract] OR "Review"[Title/Abstract] OR "reconcile*"[Title/Abstract] OR "reconciliation"[Title/Abstract] OR "therapy management"[Title/Abstract] OR "medication therapy management"[Title/Abstract] OR "follow up"[Title/Abstract] OR "follow up"[Title/Abstract] | 3,728,881 |
| #12 | | | Search: **#8 OR #9 OR #10 OR #11** Sort by: **Most Recent** | 135,436 |
| #11 | | | Search: **Medication Therapy Management[MeSH Terms]** Sort by: **Most Recent** | 2,737 |
| #10 | | | Search: **Patient Education as Topic[MeSH Terms]** Sort by: **Most Recent** | 88,530 |
| #9 | | | Search: **Directive Counseling[MeSH Terms]** Sort by: **Most Recent** | 0 |
| #8 | | | Search: **Counseling[MeSH Terms]** Sort by: **Most Recent** | 48,475 |
| #7 | | | Search: **#1 OR #2 OR #3 OR #4 OR #5 OR #6** Sort by: **Most Recent** | 959,154 |
| #6 | | | Search: **pharmac*[Title/Abstract]** Sort by: **Most Recent** | 955,041 |
| #5 | | | Search: **Students, Pharmacy[MeSH Terms]** Sort by: **Most Recent** | 4,324 |
| #4 | | | Search: **pharmacy technician[MeSH Terms]** Sort by: **Most Recent** | 883 |
| #3 | | | Search: **Pharmacists[MeSH Terms]** Filters: **Full text** Sort by: **Most Recent** | 0 |
| #2 | | | Search: **clinical pharmacy[MeSH Terms]** Filters: **Full text** Sort by: **Most Recent** | 2,267 |
| #1 | | | Search: **Pharmacy Service, Hospital[MeSH Terms]** Filters: **Full text** Sort by: **Most Recent** | 5,694 |

**CINAHL:**

| # | Query | Limiters/Expanders | Last Run Via | Results | Action |
|---|---|---|---|---|---|
| S33 | S6 AND S13 AND S20 AND S28 AND S31 | Limiters - Full Text<br>Expanders - Apply equivalent subjects<br>Search modes - Boolean/Phrase | Interface - EBSCOhost Research Databases<br>Search Screen - Advanced Search<br>Database - CINAHL Ultimate | 17 | EditS33 |
| S32 | S6 AND S13 AND S20 AND S28 AND S31 | Expanders - Apply equivalent subjects<br>Search modes - Boolean/Phrase | Interface - EBSCOhost Research Databases<br>Search Screen - Advanced Search<br>Database - CINAHL Ultimate | 17 | EditS32 |
| S31 | S29 OR S30 | Expanders - Apply equivalent subjects<br>Search modes - Boolean/Phrase | Interface - EBSCOhost Research Databases<br>Search Screen - Advanced Search<br>Database - CINAHL Ultimate | 128,337 | EditS31 |
| S30 | bahrain* OR iraq* OR kuwait* OR saudi* OR 'saudi arabia' OR oman* OR jordan* OR leban* OR iran* OR 'gaza strip' OR palestin* OR 'west bank' OR syria* OR 'syrian arab epublic' OR egypt* OR yemen* OR 'united arab emirates' OR emirat* OR 'eastern mediterranean region' OR middle east' OR 'middle-east' OR 'north africa'OR 'north-africa' OR mena OR qatar* OR algeria* OR tunisia* OR morroco* OR | Expanders - Apply equivalent subjects<br>Search modes - Boolean/Phrase | Interface - EBSCOhost Research Databases<br>Search Screen - Advanced Search<br>Database - CINAHL Ultimate | Display | EditS30 |

| | | | | | |
|---|---|---|---|---|---|
| | libya* OR djibouti* OR israel* OR malta* | | | | |
| S29 | (MM "Middle East") OR (MM "Africa, Northern") | Expanders - Apply equivalent subjects<br>Search modes - Boolean/Phrase | Interface - EBSCOhost Research Databases<br>Search Screen - Advanced Search<br>Database - CINAHL Ultimate | Display | EditS29 |
| S28 | S24 OR S27 | Expanders - Apply equivalent subjects<br>Search modes - Boolean/Phrase | Interface - EBSCOhost Research Databases<br>Search Screen - Advanced Search<br>Database - CINAHL Ultimate | 310,633 | EditS28 |
| S27 | S25 OR S26 | Expanders - Apply equivalent subjects<br>Search modes - Boolean/Phrase | Interface - EBSCOhost Research Databases<br>Search Screen - Advanced Search<br>Database - CINAHL Ultimate | 308,767 | EditS27 |
| S26 | TI (compliance OR adherence OR “medication error*” OR error* OR "medication discrepanc*" OR discrepanc*) OR AB (compliance OR adherence OR “medication error*” OR error* OR "medication discrepanc*" OR discrepanc*) OR MW (compliance OR adherence OR “medication error*” OR error* OR "medication discrepanc*" OR discrepanc*) | Expanders - Apply equivalent subjects<br>Search modes - Boolean/Phrase | Interface - EBSCOhost Research Databases<br>Search Screen - Advanced Search<br>Database - CINAHL Ultimate | Display | EditS26 |
| S25 | TI (Readmission* OR rehospitalization OR readmit* OR “hospital reutilization”) OR AB (Readmission* OR rehospitalization OR readmit* OR “hospital reutilization”) OR MW | Expanders - Apply equivalent subjects<br>Search modes - Boolean/Phrase | Interface - EBSCOhost Research Databases<br>Search Screen - Advanced | Display | EditS25 |

| | | | | | |
|---|---|---|---|---|---|
| | (Readmission* OR rehospitalization OR readmit* OR “hospital reutilization”) | | Search<br>Database - CINAHL Ultimate | | |
| S24 | S21 OR S22 OR S23 | Expanders - Apply equivalent subjects<br>Search modes - Boolean/Phrase | Interface - EBSCOhost Research Databases<br>Search Screen - Advanced Search<br>Database - CINAHL Ultimate | 49,165 | EditS24 |
| S23 | (MM "Patient Compliance+") | Expanders - Apply equivalent subjects<br>Search modes - Boolean/Phrase | Interface - EBSCOhost Research Databases<br>Search Screen - Advanced Search<br>Database - CINAHL Ultimate | Display | EditS23 |
| S22 | (MM "Medication Errors+") | Expanders - Apply equivalent subjects<br>Search modes - Boolean/Phrase | Interface - EBSCOhost Research Databases<br>Search Screen - Advanced Search<br>Database - CINAHL Ultimate | Display | EditS22 |
| S21 | (MM "Readmission") | Expanders - Apply equivalent subjects<br>Search modes - Boolean/Phrase | Interface - EBSCOhost Research Databases<br>Search Screen - Advanced Search<br>Database - CINAHL Ultimate | Display | EditS21 |
| S20 | S16 or S19 | Expanders - Apply equivalent subjects<br>Search modes - Boolean/Phrase | Interface - EBSCOhost Research Databases<br>Search Screen - Advanced Search<br>Database - CINAHL Ultimate | 115,058 | EditS20 |
| S19 | S17 OR S18 | Expanders - Apply equivalent subjects<br>Search modes - Boolean/Phrase | Interface - EBSCOhost Research Databases<br>Search Screen - Advanced | 113,409 | EditS19 |

| | | | | | |
|---|---|---|---|---|---|
| | | | Search<br>Database - CINAHL Ultimate | | |
| S18 | TI (“Transition* of care” OR "care transition* OR TOC OR “hospital to home”) OR AB (“Transition* of care” OR "care transition* OR TOC OR “hospital to home”) OR MW (“Transition* of care” OR "care transition* OR TOC OR “hospital to home”) | Expanders - Apply equivalent subjects<br>Search modes - Boolean/Phrase | Interface - EBSCOhost Research Databases<br>Search Screen - Advanced Search<br>Database - CINAHL Ultimate | Display | EditS18 |
| S17 | TI (discharge*) OR AB (discharge*) OR MW (discharge*) | Expanders - Apply equivalent subjects<br>Search modes - Boolean/Phrase | Interface - EBSCOhost Research Databases<br>Search Screen - Advanced Search<br>Database - CINAHL Ultimate | Display | EditS17 |
| S16 | S14 OR S15 | Expanders - Apply equivalent subjects<br>Search modes - Boolean/Phrase | Interface - EBSCOhost Research Databases<br>Search Screen - Advanced Search<br>Database - CINAHL Ultimate | 20,001 | EditS16 |
| S15 | (MM "Patient Discharge+") | Expanders - Apply equivalent subjects<br>Search modes - Boolean/Phrase | Interface - EBSCOhost Research Databases<br>Search Screen - Advanced Search<br>Database - CINAHL Ultimate | Display | EditS15 |
| S14 | (MM "Transitional Care") | Expanders - Apply equivalent subjects<br>Search modes - Boolean/Phrase | Interface - EBSCOhost Research Databases<br>Search Screen - Advanced Search<br>Database - CINAHL Ultimate | Display | EditS14 |

| | | | | | |
|---|---|---|---|---|---|
| S13 | S7 OR S12 | Expanders - Apply equivalent subjects<br>Search modes - Boolean/Phrase | Interface - EBSCOhost Research Databases<br>Search Screen - Advanced Search<br>Database - CINAHL Ultimate | 1,524,369 | EditS13 |
| S12 | S8 OR S9 OR S10 OR S11 | Expanders - Apply equivalent subjects<br>Search modes - Boolean/Phrase | Interface - EBSCOhost Research Databases<br>Search Screen - Advanced Search<br>Database - CINAHL Ultimate | 53,499 | EditS12 |
| S11 | (MM "Medication Management") | Expanders - Apply equivalent subjects<br>Search modes - Boolean/Phrase | Interface - EBSCOhost Research Databases<br>Search Screen - Advanced Search<br>Database - CINAHL Ultimate | Display | EditS11 |
| S10 | (MM "Medication Reconciliation") | Expanders - Apply equivalent subjects<br>Search modes - Boolean/Phrase | Interface - EBSCOhost Research Databases<br>Search Screen - Advanced Search<br>Database - CINAHL Ultimate | Display | EditS10 |
| S9 | (MM "Counseling") | Expanders - Apply equivalent subjects<br>Search modes - Boolean/Phrase | Interface - EBSCOhost Research Databases<br>Search Screen - Advanced Search<br>Database - CINAHL Ultimate | Display | EditS9 |
| S8 | (MM "Patient Education+") OR (MM "Patient Discharge Education") | Expanders - Apply equivalent subjects<br>Search modes - Boolean/Phrase | Interface - EBSCOhost Research Databases<br>Search Screen - Advanced Search<br>Database - CINAHL Ultimate | Display | EditS8 |

| | | | | | |
|---|---|---|---|---|---|
| S7 | TI (education OR counseling OR review OR reconcil* OR "therapy management") OR AB (education OR counseling OR review OR reconcil* OR "therapy management") OR MW (education OR counseling OR review OR reconcil* OR "therapy management") | Expanders - Apply equivalent subjects<br>Search modes - Boolean/Phrase | Interface - EBSCOhost Research Databases<br>Search Screen - Advanced Search<br>Database - CINAHL Ultimate | Display | EditS7 |
| S6 | S4 OR S5 | Expanders - Apply equivalent subjects<br>Search modes - Boolean/Phrase | Interface - EBSCOhost Research Databases<br>Search Screen - Advanced Search<br>Database - CINAHL Ultimate | 384,683 | EditS6 |
| S5 | S1 OR S2 OR S3 | Expanders - Apply equivalent subjects<br>Search modes - Boolean/Phrase | Interface - EBSCOhost Research Databases<br>Search Screen - Advanced Search<br>Database - CINAHL Ultimate | 11,832 | EditS5 |
| S4 | TI pharmac* OR AB pharmac* OR MW pharmac* | Expanders - Apply equivalent subjects<br>Search modes - Boolean/Phrase | Interface - EBSCOhost Research Databases<br>Search Screen - Advanced Search<br>Database - CINAHL Ultimate | 384,683 | EditS4 |
| S3 | (MM "Pharmacy Technicians") | Expanders - Apply equivalent subjects<br>Search modes - Boolean/Phrase | Interface - EBSCOhost Research Databases<br>Search Screen - Advanced Search<br>Database - CINAHL Ultimate | Display | EditS3 |
| S2 | (MM "Students, Pharmacy") | Expanders - Apply equivalent subjects<br>Search modes - Boolean/Phrase | Interface - EBSCOhost Research Databases<br>Search Screen - Advanced | Display | EditS2 |

| | | | | | |
|---|---|---|---|---|---|
| | | | Search<br>Database - CINAHL Ultimate | | |
| S1 | (MM "Pharmacists") | Expanders - Apply equivalent subjects<br>Search modes - Boolean/Phrase | Interface - EBSCOhost Research Databases<br>Search Screen - Advanced Search<br>Database - CINAHL Ultimate | Display | EditS1 |

Embase:

| | Query | Name | Last updated |
|---|---|---|---|
| | #65 | #40 AND #47 AND #52 AND #55 AND #64 | 155 |
| | #64 | #62 OR #63 | 588,246 |
| | #63 | #60 OR #61 | 539,535 |
| | #62 | #56 OR #57 OR #58 OR #59 | 192,099 |
| | #61 | readmission*:ti,ab,kw OR 're-admission*':ti,ab,kw OR rehospitalization*:ti,ab,kw OR readmitted:ti,ab,kw OR 'hospital reutilization':ti,ab,kw<br>Show full | 98,332 |
| | #60 | 'medication compliance':ti,ab,kw OR compliance:ti,ab,kw OR adherence:ti,ab,kw OR 'medication* error*':ti,ab,kw OR 'medication* discrepanc*':ti,ab,kw<br>Show full | 446,937 |
| | #59 | 'medication discrepancy'/syn | 266 |
| | #58 | 'medication compliance'/syn | 56,883 |
| | #57 | 'medication error'/syn | 26,603 |
| | #56 | 'hospital readmission'/syn | 111,203 |
| | #55 | #53 OR #54 | 1,138,019 |
| | #54 | bahrain* OR iraq* OR kuwait* OR saudi* OR 'saudi arabia' OR oman* OR jordan* OR lebanese OR lebanon OR iran* OR 'gaza strip' OR palestin* OR 'west bank' OR syria* OR 'syrian arab republic' OR egypt* OR yemen* OR 'united arab emirates' OR emirat* OR 'eastern mediterranean region' OR 'middle east' OR 'middle-east' OR 'north africa' OR 'north-africa' OR mena OR qatar* OR algeria* OR tunisia* OR morroco* OR libya* OR djibouti* OR israel* OR malta* OR 'west bank and gaza'<br>Show full | 1,656,293 |
| | #53 | 'middle eastern/north african'/syn | 11,408 |
| | #52 | #48 OR #51 | 196,461 |
| | #51 | #49 OR #50 | 193,655 |
| | #50 | 'hospital discharge'/syn | 191,712 |
| | #49 | 'transitional care'/syn | 6,994 |
| | #48 | 'transition* of care':ti,ab,kw OR 'transitional care':ti,ab,kw OR 'hospital to home':ti,ab,kw OR discharge*:ti,ab,kw | 534,867 |
| | #47 | #45 OR #46 | 3,485,645 |
| | #46 | #41 OR #42 OR #43 OR #44 | 200,468 |

Web of science:

(((((TS=(Pharmac* OR “Pharmacy Service” OR “Pharmaceutical Services”)) AND TS=(Counseling OR education OR "Medication Therapy Management" OR “therapy management”  OR review OR reconcil* )) AND TS=(Transition* OR "Transitions of care" OR "transition of care" OR "transitional care" OR "care transition" OR "hospital to home" OR discharge* )) AND TS=(Readmission* OR readmitted OR rehospitalization OR "hospital reutilization" OR error* OR "medication errors" OR discrepanc* OR Adherence OR Compliance ))) AND ALL=( bahrain* OR iraq* OR kuwait* OR saudi* OR oman* OR jordan* OR leban* OR iran* OR gaza OR palestin* OR west bank OR syria* OR  egypt* OR yemen* OR emirat* OR middle east OR north africa OR mena OR qatar* OR algeria* OR tunisia* OR morroco* OR libya* OR djibouti* OR israel* OR malta*)

**Risk of bias in included studies:**

**Risk of bias in randomized controlled trials:**

FINAL_ROB2_RCT.xlsm

**Risk of bias in quasi-experimental studies:**

**Mahroos et al (Adherence)**

# The Risk Of Bias In Non-randomized Studies – of Interventions (ROBINS-I) assessment tool

(version for cohort-type studies)

**Version 19 September 2016**

This work is licensed under a Creative Commons Attribution-NonCommercial-NoDerivatives 4.0 International License.

## ROBINS-I tool (Stage I): At protocol stage

Specify the review question

| | |
|---|---|
| Participants | Adults (≥ 18 years old) discharged from hospital (inpatient-stay or emergency visit) to home or another health care facility who receive pharmacy-managed TOC services in the MENA region |
| Experimental intervention | Pharmacy-based interventions in the context of transition of care (TOC), performed by or in coordination with pharmacy personnel (pharmacists, pharmacy students, pharmacy technicians, pharmacy interns) over the TOC continuum (i.e., at admission, during stay, at discharge, post-discharge) in the Middle East and North Africa (MENA) region. Interventions are defined as (medication reconciliation, medication review at TOC points, discharge medication counseling, post-discharge follow-up). |

| | |
|---|---|
| Comparator | Usual care (standard care) as defined in individual studies. |
| Outcomes | Healthcare utilization, medication discrepancies/medication errors, preventable adverse drug events, adherence |

List the confounding domains relevant to all or most studies

Demographic characteristics (age, gender, education level)

Comorbidities (e.g., diabetes, hypertension, cardiovascular disease, comorbidity severity index)

History of health care utilization (history of prior hospital admissions or emergency visits)

Medication-related risk (polypharmacy, number of medications at discharge, high-risk medications at discharge)

List co-interventions that could be different between intervention groups and that could impact on outcomes

Receiving additional care by other Healthcare professionals (concomitant to the intervention), e.g., education by nurses, more frequent clinic visits.

## ROBINS-I tool (Stage II): For each study

Specify a target randomized trial specific to the study

Design Individually randomized / Cluster randomized / Matched (e.g. cross-over)

| | |
|---|---|
| Participants | Adults discharged from hospital on warfarin for follow up at outpatient settings |
| Experimental intervention | Pharmacist-managed anticoagulation clinic for follow-up and education post-hospital discharge |
| Comparator | Usual care-standard care (follow up delivered by physicians/ nurses) |

Is your aim for this study…?

√☐ to assess the effect of *assignment to* intervention

☐ to assess the effect of *starting and adhering to* intervention

Specify the outcome

Specify which outcome is being assessed for risk of bias (typically from among those earmarked for the Summary of Findings table). Specify whether this is a proposed benefit or harm of intervention.

Patients' adherence to medications

Specify the numerical result being assessed

In case of multiple alternative analyses being presented, specify the numeric result (e.g. RR = 1.52 (95% CI 0.83 to 2.77) and/or a reference (e.g. to a table, figure or paragraph) that uniquely defines the result being assessed.

Patient adherence to warfarin at 30-60 and 90-days:

(Percentage of patients with good adherence):

C:100%-100%-100%

I: 87.0%- 87.0%- 82.6%, respectively (unadjusted)

Preliminary consideration of confounders

Complete a row for each important confounding domain (i) listed in the review protocol; and (ii) relevant to the setting of this particular study, or which the study authors identified as potentially important.

*"Important" confounding domains are those for which, in the context of this study, adjustment is expected to lead to a clinically important change in the estimated effect of the intervention. "Validity" refers to whether the confounding variable or variables fully measure the domain, while "reliability" refers to the precision of the measurement (more measurement error means less reliability).*

| **(i) Confounding domains listed in the review protocol** | | | | |
|---|---|---|---|---|
| Confounding domain | Measured variable(s) | Is there evidence that controlling for this variable was unnecessary?* | Is the confounding domain measured validly and reliably by this variable (or these variables)? | OPTIONAL: Is failure to adjust for this variable (alone) expected to favour the |

| | | | | |
|---|---|---|---|---|
| | | | | experimental intervention or the comparator? |
| Demographics | Age, gender, BMI, educational level | No | NI (authors did not describe method of data collection) | |
| Comorbidities | Number concomitant chronic diseases, drug allergy | No | NI (authors did not describe method of data collection) | |
| Medication-related risk (polypharmacy, number of medications at discharge, high-risk medications at discharge) | Not measured | No | No | |

| **(ii) Additional confounding domains relevant to the setting of this particular study, or which the study authors identified as important** | | | | |
|---|---|---|---|---|
| Confounding domain | Measured variable(s) | Is there evidence that controlling for this variable was unnecessary?* | Is the confounding domain measured validly and reliably by this variable (or these variables)? | OPTIONAL: Is failure to adjust for this variable (alone) expected to favour the experimental intervention or the comparator? |
| Social habits | Smoking status | No. Smoking can affect INR control in warfarin-treated patients and thus may affect adherence to warfarin | NI (authors did not describe method of data collection) | |

* In the context of a particular study, variables can be demonstrated not to be confounders and so not included in the analysis: (a) if they are not predictive of the outcome; (b) if they are not predictive of intervention; or (c) because adjustment makes no or minimal difference to the estimated effect of the primary parameter. Note that “no statistically significant association” is not the same as “not predictive”.

Preliminary consideration of co-interventions

Complete a row for each important co-intervention (i) listed in the review protocol; and (ii) relevant to the setting of this particular study, or which the study authors identified as important.

*“Important” co-interventions are those for which, in the context of this study, adjustment is expected to lead to a clinically important change in the estimated effect of the intervention.*

| **(i) Co-interventions listed in the review protocol** | | |
|---|---|---|
| Co-intervention | Is there evidence that controlling for this co-intervention was unnecessary (e.g. because it was not administered)? | Is presence of this co-intervention likely to favour outcomes in the experimental intervention or the comparator |
| Receiving additional care by other Healthcare professionals (concomitant to the intervention), e.g., education by nurses, more frequent clinic visits) | No information | Favour experimental / Favour comparator / No information |

| **(ii) Additional co-interventions relevant to the setting of this particular study, or which the study authors identified as important** | | |
|---|---|---|
| Co-intervention | Is there evidence that controlling for this co-intervention was unnecessary (e.g. because it was not administered)? | Is presence of this co-intervention likely to favour outcomes in the experimental intervention or the comparator |
| None | - | Favour experimental / Favour comparator / No information |

Risk of bias assessment. Responses underlined in green are potential markers for low risk of bias, and responses in red are potential markers for a risk of bias. Where questions relate only to sign posts to other questions, no formatting is used.

| Signalling questions | Description | Response options |
|---|---|---|
| **Bias due to confounding** | | |
| 1.1 Is there potential for confounding of the effect of intervention in this study?<br><br>**If N/PN to 1.1:** the study can be considered to be at low risk of bias due to confounding and no further signalling questions need be considered | | Y / PY / PN / N |
| **If Y/PY to 1.1**: determine whether there is a need to assess time-varying confounding: | Baseline confounding occurs when one or more pre-intervention prognostic factors predict the intervention received at start of follow up.<br><br>The authors did not report the criteria of allocating participants to intervention or control group.<br><br>The possibility that patients were allocated to either group based on baseline confounders, that could also affect adherence (outcome) can't be ruled out. Although "baseline confounding" in this context is more relevant to observational/non-experimental studies but it should be considered in this context. | |
| 1.2. Was the analysis based on splitting participants' follow up time according to intervention received? | **N/PN** | NA / Y / PY / PN / N / NI |

| | | |
|---|---|---|
| **If N/PN**, answer questions relating to baseline confounding (1.4 to 1.6)<br><br>**If Y/PY**, go to question 1.3. | | |
| 1.3. Were intervention discontinuations or switches likely to be related to factors that are prognostic for the outcome?<br><br>**If N/PN**, answer questions relating to baseline confounding (1.4 to 1.6)<br><br>**If Y/PY**, answer questions relating to both baseline and time-varying confounding (1.7 and 1.8) | | NA / Y / PY / PN / N / NI |

| | | |
|---|---|---|
| **Questions relating to baseline confounding only** | | |
| 1.4. Did the authors use an appropriate analysis method that controlled for all the important confounding domains? | The authors did not adjust the outcome for any of the potential confounders. | NA / Y / PY / PN / N / NI |
| 1.5. **If Y/PY to 1.4**: Were confounding domains that were controlled for measured validly and reliably by the variables available in this study? | | NA / Y / PY / PN / N / NI |
| 1.6. Did the authors control for any post-intervention variables that could have been affected by the intervention? | | NA / Y / PY / PN / N / NI |
| **Questions relating to baseline and time-varying confounding** | | |

| | | |
|---|---|---|
| 1.7. Did the authors use an appropriate analysis method that controlled for all the important confounding domains and for time-varying confounding? | | NA / Y / PY / PN / N / NI |
| 1.8. **If Y/PY to 1.7**: Were confounding domains that were controlled for measured validly and reliably by the variables available in this study? | | NA / Y / PY / PN / N / NI |
| **Risk of bias judgement** | Serious | Low / Moderate / Serious / Critical / NI |
| Optional: What is the predicted direction of bias due to confounding? | | Favours experimental / Favours comparator / Unpredictable |

| **Bias in selection of participants into the study** | | |
|---|---|---|
| 2.1. Was selection of participants into the study (or into the analysis) based on participant characteristics observed after the start of intervention?<br><br>**If N/PN to 2.1:** go to 2.4 | Selection of participants was done at baseline before allocation participants to groups. | Y / PY / PN / N / NI |
| 2.2. **If Y/PY to 2.1**: Were the post-intervention variables that influenced selection likely to be associated with intervention? | | NA / Y / PY / PN / N / NI |
| 2.3 **If Y/PY to 2.2**: Were the post-intervention variables that influenced selection likely to be influenced by the outcome or a cause of the outcome? | | NA / Y / PY / PN / N / NI |

| | | |
|---|---|---|
| 2.4. Do start of follow-up and start of intervention coincide for most participants? | | Y / PY / PN / N / NI |
| 2.5. **If Y/PY to 2.2 and 2.3, or N/PN to 2.4**: Were adjustment techniques used that are likely to correct for the presence of selection biases? | N/A | NA / Y / PY / PN / N / NI |
| **Risk of bias judgement** | Low | Low / Moderate / Serious / Critical / NI |
| Optional: What is the predicted direction of bias due to selection of participants into the study? | | Favours experimental / Favours comparator / Towards null /Away from null / Unpredictable |

| **Bias in classification of interventions** | | |
|---|---|---|
| 3.1 Were intervention groups clearly defined? | Intervention and usual care were described (interventions received in each group). Criteria for considering individuals to have received each intervention was not clarified. | Y / PY / PN / N / NI |
| 3.2 Was the information used to define intervention groups recorded at the start of the intervention? | Yes. But the criteria on which patients were allocated to each group was not described and the details of interventions received by usual care was barely described. However, this is not captured by this question. (Limitation of using this tool for quasi-experimental studies). | Y / PY / PN / N / NI |

| | | |
|---|---|---|
| 3.3 Could classification of intervention status have been affected by knowledge of the outcome or risk of the outcome? | This is less relevant in a quasi-experimental study where participants were allocated to intervention before knowledge of the outcome | Y / PY / PN / N / NI |
| **Risk of bias judgement** | Low | Low / Moderate / Serious / Critical / NI |
| Optional: What is the predicted direction of bias due to classification of interventions? | | Favours experimental / Favours comparator / Towards null /Away from null / Unpredictable |

| **Bias due to deviations from intended interventions** | | |
|---|---|---|
| **If your aim for this study is to assess the effect of assignment to intervention, answer questions 4.1 and 4.2** | | |
| 4.1. Were there deviations from the intended intervention beyond what would be expected in usual practice? | | Y / PY / PN / N / NI |
| 4.2. **If Y/PY to 4.1**: Were these deviations from intended intervention unbalanced between groups *and* likely to have affected the outcome? | | NA / Y / PY / PN / N / NI |
| **If your aim for this study is to assess the effect of starting and adhering to intervention, answer questions 4.3 to 4.6** | | |
| 4.3. Were important co-interventions balanced across intervention groups? | | Y / PY / PN / N / NI |
| 4.4. Was the intervention implemented successfully for most participants? | | Y / PY / PN / N / NI |

| | | |
|---|---|---|
| 4.5. Did study participants adhere to the assigned intervention regimen? | | Y / PY / PN / N / NI |
| 4.6. **If N/PN to 4.3, 4.4 or 4.5**: Was an appropriate analysis used to estimate the effect of starting and adhering to the intervention? | | NA / Y / PY / PN / N / NI |
| **Risk of bias judgement** | Low | Low / Moderate / Serious / Critical / NI |
| Optional: What is the predicted direction of bias due to deviations from the intended interventions? | | Favours experimental / Favours comparator / Towards null /Away from null / Unpredictable |

| **Bias due to missing data** | | |
|---|---|---|
| 5.1 Were outcome data available for all, or nearly all, participants? | Outcome reported for all enrolled subjects in both groups | Y / PY / PN / N / NI |
| 5.2 Were participants excluded due to missing data on intervention status? | | Y / PY / PN / N / NI |
| 5.3 Were participants excluded due to missing data on other variables needed for the analysis? | | Y / PY / PN / N / NI |
| 5.4 **If PN/N to 5.1, or Y/PY to 5.2 or 5.3**: Are the proportion of participants and reasons for missing data similar across interventions? | | NA / Y / PY / PN / N / NI |

| | | |
|---|---|---|
| 5.5 **If PN/N to 5.1, or Y/PY to 5.2 or 5.3**: Is there evidence that results were robust to the presence of missing data? | | NA / Y / PY / PN / N / NI |
| **Risk of bias judgement** | Low | Low / Moderate / Serious / Critical / NI |
| Optional: What is the predicted direction of bias due to missing data? | | Favours experimental / Favours comparator / Towards null /Away from null / Unpredictable |

| **Bias in measurement of outcomes** | | |
|---|---|---|
| 6.1 Could the outcome measure have been influenced by knowledge of the intervention received? | Method of assessing outcome was not reported. However, upon contacting the second author, they clarified that adherence was assessed by asking patients about how many doses were missed during last month.<br><br>Detection bias can arise since the outcome assessors (patients in this self-reported outcome) are aware of intervention status. Bias may also arise from using different methods or intensities of questioning patients about adherence (in the two groups) since the outcome assessors were not blinded. | Y / PY / PN / N / NI |
| 6.2 Were outcome assessors aware of the intervention received by study participants? | Patients (un-blinded) | Y / PY / PN / N / NI |
| 6.3 Were the methods of outcome assessment comparable across intervention groups? | Same criteria at same time points. However, bias may arise from using different methods or intensities of questioning patients about adherence (in the two groups) since the outcome assessors were not blinded. | Y / PY / PN / N / NI |

| | | |
|---|---|---|
| 6.4 Were any systematic errors in measurement of the outcome related to intervention received? | | Y / PY / PN / N / NI |
| **Risk of bias judgement** | Serious | Low / Moderate / Serious / Critical / NI |
| Optional: What is the predicted direction of bias due to measurement of outcomes? | | Favours experimental / Favours comparator / Towards null /Away from null / Unpredictable |

| **Bias in selection of the reported result** | | |
|---|---|---|
| Is the reported effect estimate likely to be selected, on the basis of the results, from... | The reporting was poor. Authors did not clarify outcomes in methods section. But it is unlikely that they selected it from multiple measurement methods or analyses. | |
| 7.1. ... multiple outcome *measurements* within the outcome domain? | | Y / PY / PN / N / NI |
| 7.2 ... multiple *analyses* of the intervention-outcome relationship? | | Y / PY / PN / N / NI |
| 7.3 ... different *subgroups*? | | Y / PY / PN / N / NI |
| **Risk of bias judgement** | Low | Low / Moderate / Serious / Critical / NI |
| Optional: What is the predicted direction of bias due to selection of the reported result? | | Favours experimental / Favours comparator / Towards null /Away from null / Unpredictable |

| Overall bias | | |
|---|---|---|
| **Risk of bias judgement** | **Serious** | Low / Moderate / Serious / Critical / NI |
| Optional: What is the overall predicted direction of bias for this outcome? | | Favours experimental / Favours comparator / Towards null /Away from null / Unpredictable |

This work is licensed under a Creative Commons Attribution-NonCommercial-NoDerivatives 4.0 International License.

**El-Hajj et al (Healthcare utilization):**

**The Risk Of Bias In Non-randomized Studies – of Interventions (ROBINS-I) assessment tool**

(version for cohort-type studies)

**Version 19 September 2016**

This work is licensed under a Creative Commons Attribution-NonCommercial-NoDerivatives 4.0 International License.

**ROBINS-I tool (Stage I): At protocol stage**

Specify the review question

| | |
|---|---|
| Participants | Adults (≥ 18 years old) discharged from hospital (inpatient-stay or emergency visit) to home or another health care facility who receive pharmacy-managed TOC services in the MENA region |
| Experimental intervention | Pharmacy-based interventions in the context of transition of care (TOC), performed by or in coordination with pharmacy personnel (pharmacists, pharmacy students, pharmacy technicians, pharmacy interns) over the TOC continuum (i.e., at admission, during stay, at discharge, post-discharge) in the Middle East and North Africa (MENA) region. Interventions are defined as (medication reconciliation, medication review at TOC points, discharge medication counseling, post-discharge follow-up). |
| Comparator | Usual care (standard care) as defined in individual studies. |
| Outcomes | Healthcare utilization, medication discrepancies/medication errors, preventable adverse drug events, adherence |

**List the confounding domains relevant to all or most studies**

| |
|---|
| Demographic characteristics (age, gender, education level)<br>Comorbidities (e.g., diabetes, hypertension, cardiovascular disease, comorbidity severity index)<br>History of health care utilization (history of prior hospital admissions or emergency visits)<br>Medication-related risk (polypharmacy, number of medications at discharge, high-risk medications at discharge) |

**List co-interventions that could be different between intervention groups and that could impact on outcomes**

| |
|---|
| Receiving additional care by other Healthcare professionals (concomitant to the intervention), e.g., education by nurses, more frequent clinic visits |

ROBINS-I tool (Stage II): For each study

Specify a target randomized trial specific to the study

| | |
|---|---|
| Design | Individually randomized / Cluster randomized / Matched (e.g. cross-over) |
| Participants | Adults with acute coronary syndrome discharged from hospital |
| Experimental intervention | Structured pharmacist intervention at and post-discharge (reconciliation, education, prescription review, post-discharge phone calls) |
| Comparator | Usual care-standard care (either standard routine pharmacist interventions at discharge or no pharmacist involvement) |

**Is your aim for this study…?**

- √ to assess the effect of *assignment to* intervention
- ☐ to assess the effect of *starting and adhering to* intervention

**Specify the outcome**

Specify which outcome is being assessed for risk of bias (typically from among those earmarked for the Summary of Findings table). Specify whether this is a proposed benefit or harm of intervention.

Healthcare utilization. All cause hospitalization at 6 months.

Specify the numerical result being assessed

In case of multiple alternative analyses being presented, specify the numeric result (e.g. RR = 1.52 (95% CI 0.83 to 2.77) and/or a reference (e.g. to a table, figure or paragraph) that uniquely defines the result being assessed.

**All-cause hospitalizations**
Usual care vs. intervention (reference): adjusted OR 1.701, (95%CI 0.888-3.257), P=0.109
Control vs. intervention (reference): adjusted OR 1.744 (95% CI 0.876-3.474), P= 0.114
Usual care+ control arm vs. intervention (reference): adjusted OR
1.719 (95% 0.941-3.138), P= 0.07

Preliminary consideration of confounders

Complete a row for each important confounding domain (i) listed in the review protocol; and (ii) relevant to the setting of this particular study, or which the study authors identified as potentially important.

"Important" confounding domains are those for which, in the context of this study, adjustment is expected to lead to a clinically important change in the estimated effect of the intervention. "Validity" refers to whether the confounding variable or variables fully measure the domain, while "reliability" refers to the precision of the measurement (more measurement error means less reliability).

| **(i) Confounding domains listed in the review protocol** | | | | |
|---|---|---|---|---|
| Confounding domain | Measured variable(s) | Is there evidence that controlling for this variable was unnecessary?* | Is the confounding domain measured validly and reliably by this variable (or these variables)? | OPTIONAL: Is failure to adjust for this variable (alone) expected to favour the experimental intervention or the comparator? |
| Demographics | Age, gender, BMI | No | Yes | |
| Comorbidities | Number concomitant chronic diseases, drug allergy | No | Yes | |
| Medication-related risk (polypharmacy, number of medications at discharge, high-risk medications at discharge) | Not measured | No | No | |

| | | | | |
|---|---|---|---|---|
| History of healthcare utilization (history of prior hospital admissions or emergency visits) | Not measured | No | No | |

| (ii) Additional confounding domains relevant to the setting of this particular study, or which the study authors identified as important | | | | |
|---|---|---|---|---|
| Confounding domain | Measured variable(s) | Is there evidence that controlling for this variable was unnecessary?* | Is the confounding domain measured validly and reliably by this variable (or these variables)? | OPTIONAL: Is failure to adjust for this variable (alone) expected to favour the experimental intervention or the comparator? |
| Concomitant procedures | PCI (Percutaneous Coronary Intervention) done or not | No | Yes | |

* In the context of a particular study, variables can be demonstrated not to be confounders and so not included in the analysis: (a) if they are not predictive of the outcome; (b) if they are not predictive of intervention; or (c) because adjustment makes no or minimal difference to the estimated effect of the primary parameter. Note that "no statistically significant association" is not the same as "not predictive".Preliminary consideration of co-interventions

Complete a row for each important co-intervention (i) listed in the review protocol; and (ii) relevant to the setting of this particular study, or which the study authors identified as important.

"Important" co-interventions are those for which, in the context of this study, adjustment is expected to lead to a clinically important change in the estimated effect of the intervention.

| (i) Co-interventions listed in the review protocol | | |
|---|---|---|
| Co-intervention | Is there evidence that controlling for this co-intervention was unnecessary (e.g. because it was not administered)? | Is presence of this co-intervention likely to favour outcomes in the experimental intervention or the comparator |

| | | |
|---|---|---|
| Receiving additional care by other Healthcare professionals (concomitant to the intervention), e.g., education by nurses, more frequent clinic visits) | No information | Favour experimental / Favour comparator / No information |

| (ii) Additional co-interventions relevant to the setting of this particular study, or which the study authors identified as important | | |
|---|---|---|
| Co-intervention | Is there evidence that controlling for this co-intervention was unnecessary (e.g. because it was not administered)? | Is presence of this co-intervention likely to favour outcomes in the experimental intervention or the comparator |
| None | - | Favour experimental / Favour comparator / No information |

Risk of bias assessment. Responses underlined in green are potential markers for low risk of bias, and responses in red are potential markers for a risk of bias. Where questions relate only to sign posts to other questions, no formatting is used.

| Signalling questions | Description | Response options |
|---|---|---|
| **Bias due to confounding** | | |
| 1.1 Is there potential for confounding of the effect of intervention in this study?<br>**If N/PN to 1.1:** the study can be considered to be at low risk of bias due to confounding and no further signalling questions need be considered | | Y / PY / PN / N |

| | | |
|---|---|---|
| **If Y/PY to 1.1**: determine whether there is a need to assess time-varying confounding: | Baseline confounding occurs when one or more pre-intervention prognostic factors predict the intervention received at start of follow up.<br>The study adopted "an inherent" process of randomly allocating patients to groups (intervention, usual care, control).<br>"First, consenting patients who happen to be allocated to the teams that have the intervention clinical pharmacists will be naturally considered<br>in the intervention arm. Second, consenting patients who happen to be allocated to the teams that have the non-intervention clinical pharmacists will be considered in the usual care arm. Finally, consenting patients who are discharged on weekends and after the clinical pharmacists' working hours will be naturally randomised into<br>the control arm". The possibility that allocation of patients to different teams according to their complexity (baseline characteristics) or that those discharged over the weekend may be less complex and don't need intervention can't be ruled out. Although "baseline confounding" in this context is more relevant to observational/non-experimental studies but it should be considered in this context. But time varying confounding, which occurs when follow-up time is split according to the intervention received is not unlikely a concern is a prospective quasi-randomized study. | |
| 1.2. Was the analysis based on splitting participants' follow up time according to intervention received?<br>**If N/PN**, answer questions relating to baseline confounding (1.4 to 1.6)<br>**If Y/PY**, go to question 1.3. | **N/PN** | NA / Y / PY / PN / N / NI |

| | | |
|---|---|---|
| 1.3. Were intervention discontinuations or switches likely to be related to factors that are prognostic for the outcome?<br>**If N/PN**, answer questions relating to baseline confounding (1.4 to 1.6)<br>**If Y/PY**, answer questions relating to both baseline and time-varying confounding (1.7 and 1.8) | | NA / Y / PY / PN / N / NI |
| **Questions relating to baseline confounding only** | | |
| 1.4. Did the authors use an appropriate analysis method that controlled for all the important confounding domains? | Logistic regression analysis was used to adjust for baseline imbalances Outcome was adjusted for age, gender, PCI, CAD, diabetes, obesity, type of ACS, hypertension, hyperlipidemia), but authors did not consider domains related to prior utilization of healthcare (e.g., frequent readmission) and complexity of medication regimens (e.g., polypharmacy), and they did not adjust for these domains.<br>The baseline confounders could affect allocation to intervention and the outcome of healthcare utilization. Confounders controlled for were objective data collected through medical chart review and patient interview. (Valid and reliable) | NA / Y / PY / PN / N / NI |
| 1.5. **If Y/PY to 1.4**: Were confounding domains that were controlled for measured validly and reliably by the variables available in this study? | | NA / Y / PY / PN / N / NI |
| 1.6. Did the authors control for any post-intervention variables that could have been affected by the intervention? | | NA / Y / PY / PN / N / NI |
| **Questions relating to baseline and time-varying confounding** | | |
| 1.7. Did the authors use an appropriate analysis method that controlled for all the important confounding domains and for time-varying confounding? | | NA / Y / PY / PN / N / NI |

| | | |
|---|---|---|
| 1.8. **If Y/PY to 1.7**: Were confounding domains that were controlled for measured validly and reliably by the variables available in this study? | | NA / Y / PY / PN / N / NI |
| **Risk of bias judgement** | Serious | Low / Moderate / Serious / Critical / NI |
| Optional: What is the predicted direction of bias due to confounding? | | Favours experimental / Favours comparator / Unpredictable |

| **Bias in selection of participants into the study** | | |
|---|---|---|
| 2.1. Was selection of participants into the study (or into the analysis) based on participant characteristics observed after the start of intervention?<br>**If N/PN to 2.1:** go to 2.4<br>2.2. **If Y/PY to 2.1**: Were the post-intervention variables that influenced selection likely to be associated with intervention?<br>2.3 **If Y/PY to 2.2**: Were the post-intervention variables that influenced selection likely to be influenced by the outcome or a cause of the outcome? | Selection of participants was done at baseline before allocation participants to groups. | Y / PY / PN / N / NI<br><br>NA / Y / PY / PN / N / NI<br><br>NA / Y / PY / PN / N / NI |
| 2.4. Do start of follow-up and start of intervention coincide for most participants? | | Y / PY / PN / N / NI |
| 2.5. **If Y/PY to 2.2 and 2.3, or N/PN to 2.4**: Were adjustment techniques used that are likely to correct for the presence of selection biases? | N/A | NA / Y / PY / PN / N / NI |
| **Risk of bias judgement** | Low | Low / Moderate / Serious / Critical / NI |

| | | |
|---|---|---|
| Optional: What is the predicted direction of bias due to selection of participants into the study? | | Favours experimental / Favours comparator / Towards null /Away from null / Unpredictable |

| Bias in classification of interventions | | |
|---|---|---|
| 3.1 Were intervention groups clearly defined? | Intervention, usual care, and control arms were reasonably described (interventions received in each group). Criteria for considering individuals to have received each intervention was clear. | Y / PY / PN / N / NI |
| 3.2 Was the information used to define intervention groups recorded at the start of the intervention? | Yes. They were recorded at protocol stage before the start of the intervention. | Y / PY / PN / N / NI |
| 3.3 Could classification of intervention status have been affected by knowledge of the outcome or risk of the outcome? | This is less relevant in a quasi-experimental study where participants were allocated to intervention before knowledge of the outcome | Y / PY / PN / N / NI |
| **Risk of bias judgement** | Low | Low / Moderate / Serious / Critical / NI |
| Optional: What is the predicted direction of bias due to classification of interventions? | | Favours experimental / Favours comparator / Towards null /Away from null / Unpredictable |

| Bias due to deviations from intended interventions | | |
|---|---|---|
| **If your aim for this study is to assess the effect of assignment to intervention, answer questions 4.1 and 4.2** | | |
| 4.1. Were there deviations from the intended intervention beyond what would be expected in usual practice? | | Y / PY / PN / N / NI |

| | | |
|---|---|---|
| 4.2. **If Y/PY to 4.1**: Were these deviations from intended intervention unbalanced between groups *and* likely to have affected the outcome? | | NA / Y / PY / PN / N / NI |
| **If your aim for this study is to assess the effect of starting and adhering to intervention, answer questions 4.3 to 4.6** | | |
| 4.3. Were important co-interventions balanced across intervention groups? | | Y / PY / PN / N / NI |
| 4.4. Was the intervention implemented successfully for most participants? | | Y / PY / PN / N / NI |
| 4.5. Did study participants adhere to the assigned intervention regimen? | | Y / PY / PN / N / NI |
| 4.6. **If N/PN to 4.3, 4.4 or 4.5**: Was an appropriate analysis used to estimate the effect of starting and adhering to the intervention? | | NA / Y / PY / PN / N / NI |
| **Risk of bias judgement** | Low | Low / Moderate / Serious / Critical / NI |
| Optional: What is the predicted direction of bias due to deviations from the intended interventions? | | Favours experimental / Favours comparator / Towards null /Away from null / Unpredictable |

| **Bias due to missing data** | | |
|---|---|---|
| 5.1 Were outcome data available for all, or nearly all, participants? | Outcomes reported for all enrolled subjects and "intention to treat" analysis. | Y / PY / PN / N / NI |
| 5.2 Were participants excluded due to missing data on intervention status? | | Y / PY / PN / N / NI |
| 5.3 Were participants excluded due to missing data on other variables needed for the analysis? | | Y / PY / PN / N / NI |
| 5.4 **If PN/N to 5.1, or Y/PY to 5.2 or 5.3**: Are the proportion of participants and reasons for missing data similar across interventions? | | NA / Y / PY / PN / N / NI |

| | | |
|---|---|---|
| 5.5 **If PN/N to 5.1, or Y/PY to 5.2 or 5.3**: Is there evidence that results were robust to the presence of missing data? | | NA / Y / PY / PN / N / NI |
| **Risk of bias judgement** | Low | Low / Moderate / Serious / Critical / NI |
| Optional: What is the predicted direction of bias due to missing data? | | Favours experimental / Favours comparator / Towards null /Away from null / Unpredictable |

| **Bias in measurement of outcomes** | | |
|---|---|---|
| 6.1 Could the outcome measure have been influenced by knowledge of the intervention received? | Outcome assessors were blinded to the intervention received and reported readmissions from medical records at same time points /same definition | Y / PY / PN / N / NI |
| 6.2 Were outcome assessors aware of the intervention received by study participants? | | Y / PY / PN / N / NI |
| 6.3 Were the methods of outcome assessment comparable across intervention groups? | | Y / PY / PN / N / NI |
| 6.4 Were any systematic errors in measurement of the outcome related to intervention received? | Outcome was assessed only by capturing medical records which may miss some readmissions, but this applies to all groups. | Y / PY / PN / N / NI |
| **Risk of bias judgement** | Low | Low / Moderate / Serious / Critical / NI |
| Optional: What is the predicted direction of bias due to measurement of outcomes? | | Favours experimental / Favours comparator / Towards null /Away from null / Unpredictable |

| Bias in selection of the reported result | | |
|---|---|---|
| Is the reported effect estimate likely to be selected, on the basis of the results, from...<br>7.1. ... multiple outcome *measurements* within the outcome domain? | Authors planned to check readmission at 3,6 and 12 months but reported on data at 6 months | Y / PY / PN / N / NI |
| 7.2 ... multiple *analyses* of the intervention-outcome relationship? | The protocol stated that (Two sensitivity analyses will be performed one using multiple imputations by chained equations for the missing data and the other using per protocol methodology) but results not presented. | Y / PY / PN / N / NI |
| 7.3 ... different *subgroups*? | | Y / PY / PN / N / NI |
| **Risk of bias judgement** | Serious | Low / Moderate / Serious / Critical / NI |
| Optional: What is the predicted direction of bias due to selection of the reported result? | | Favours experimental / Favours comparator / Towards null /Away from null / Unpredictabl e |

| Overall bias | | |
|---|---|---|
| **Risk of bias judgement** | **Serious** | Low / Moderate / Serious / Critical / NI |

| | | | |
|---|---|---|---|
| | Optional: What is the overall predicted direction of bias for this outcome? | | Favours experimental / Favours comparator / Towards null /Away from null / Unpredictable |

This work is licensed under a Creative Commons Attribution-NonCommercial-NoDerivatives 4.0 International License.

**El-Hajj et al (Adherence):**

**The Risk Of Bias In Non-randomized Studies – of Interventions (ROBINS-I) assessment tool**

**(version for cohort-type studies)**

**Version 19 September 2016**

This work is licensed under a Creative Commons Attribution-NonCommercial-NoDerivatives 4.0 International License.

ROBINS-I tool (Stage I): At protocol stage

Specify the review question

| | |
|---|---|
| Participants | Adults ($\geq$ 18 years old) discharged from hospital (inpatient-stay or emergency visit) to home or another health care facility who receive pharmacy-managed TOC services in the MENA region |
| Experimental intervention | Pharmacy-based interventions in the context of transition of care (TOC), performed by or in coordination with pharmacy personnel (pharmacists, pharmacy students, pharmacy technicians, pharmacy interns) over the TOC continuum (i.e., at admission, during stay, at discharge, post-discharge) in the Middle East and North Africa (MENA) region. Interventions are defined as (medication reconciliation, medication review at TOC points, discharge medication counseling, post-discharge follow-up). |
| Comparator | Usual care (standard care) as defined in individual studies. |
| Outcomes | Healthcare utilization, medication discrepancies/medication errors, preventable adverse drug events, adherence |

**List the confounding domains relevant to all or most studies**

| |
|---|
| Demographic characteristics (age, gender, education level)<br>Comorbidities (e.g., diabetes, hypertension, cardiovascular disease, comorbidity severity index)<br>History of health care utilization (history of prior hospital admissions or emergency visits)<br>Medication-related risk (polypharmacy, number of medications at discharge, high-risk medications at discharge) |

List co-interventions that could be different between intervention groups and that could impact on outcomes

| |
|---|
| Receiving additional care by other Healthcare professionals (concomitant to the intervention), e.g., education by nurses, more frequent clinic visits. |

ROBINS-I tool (Stage II): For each study

| | |
|---|---|
| Design | Individually randomized / Cluster randomized / Matched (e.g. cross-over) |
| Participants | Adults with acute coronary syndrome discharged from hospital |
| Experimental intervention | Structured pharmacist intervention at and post-discharge (reconciliation, education, prescription review, post-discharge phone calls) |
| Comparator | Usual care-standard care (either standard routine pharmacist interventions at discharge or no pharmacist involvement) |

Specify a target randomized trial specific to the study

Is your aim for this study…?

√ to assess the effect of *assignment to* intervention

☐ to assess the effect of *starting and adhering to* intervention

Specify the outcome

Specify which outcome is being assessed for risk of bias (typically from among those earmarked for the Summary of Findings table). Specify whether this is a proposed benefit or harm of intervention.

| |
|---|
| Adherence to medications |

Specify the numerical result being assessed

In case of multiple alternative analyses being presented, specify the numeric result (e.g. RR = 1.52 (95% CI 0.83 to 2.77) and/or a reference (e.g. to a table, figure or paragraph) that uniquely defines the result being assessed.

| |
|---|
| Adherence to evidence-based secondary prevention therapy (Proportion of days covered (PDC), prescription refill records at outpatient pharmacy), number of patients (percentage) with ADC>75%:<br>I: 65 (60.7%) vs. Usual care: 72 (60.0%) vs. C: 68 (50.0%), P= 0.156 (unadjusted) |

Preliminary consideration of confounders

Complete a row for each important confounding domain (i) listed in the review protocol; and (ii) relevant to the setting of this particular study, or which the study authors identified as potentially important.

"Important" confounding domains are those for which, in the context of this study, adjustment is expected to lead to a clinically important change in the estimated effect of the intervention. "Validity" refers to whether the confounding variable or variables fully measure the domain, while "reliability" refers to the precision of the measurement (more measurement error means less reliability).

| **(i) Confounding domains listed in the review protocol** | | | | |
|---|---|---|---|---|
| Confounding domain | Measured variable(s) | Is there evidence that controlling for this variable was unnecessary?* | Is the confounding domain measured validly and reliably by this variable (or these variables)? | OPTIONAL: Is failure to adjust for this variable (alone) expected to favour the experimental intervention or the comparator? |
| Demographics | Age, gender, BMI | No | Yes | |
| Comorbidities | Number concomitant chronic diseases, drug allergy | No | Yes | |
| Medication-related risk (polypharmacy, number of medications at discharge, high-risk medications at discharge) | Not measured | No | No | |

| **(ii) Additional confounding domains relevant to the setting of this particular study, or which the study authors identified as important** |
|---|

| Confounding domain | Measured variable(s) | Is there evidence that controlling for this variable was unnecessary?* | Is the confounding domain measured validly and reliably by this variable (or these variables)? | OPTIONAL: Is failure to adjust for this variable (alone) expected to favour the experimental intervention or the comparator? |
|---|---|---|---|---|

* In the context of a particular study, variables can be demonstrated not to be confounders and so not included in the analysis: (a) if they are not predictive of the outcome; (b) if they are not predictive of intervention; or (c) because adjustment makes no or minimal difference to the estimated effect of the primary parameter. Note that "no statistically significant association" is not the same as "not predictive".Preliminary consideration of co-interventions

Complete a row for each important co-intervention (i) listed in the review protocol; and (ii) relevant to the setting of this particular study, or which the study authors identified as important.

"Important" co-interventions are those for which, in the context of this study, adjustment is expected to lead to a clinically important change in the estimated effect of the intervention.

| **(i) Co-interventions listed in the review protocol** | | |
|---|---|---|
| Co-intervention | Is there evidence that controlling for this co-intervention was unnecessary (e.g. because it was not administered)? | Is presence of this co-intervention likely to favour outcomes in the experimental intervention or the comparator |
| Receiving additional care by other Healthcare professionals (concomitant to the intervention), e.g., education by nurses, more frequent clinic visits) | No information | Favour experimental / Favour comparator / No information |

| **(ii) Additional co-interventions relevant to the setting of this particular study, or which the study authors identified as important** | | |
|---|---|---|
| Co-intervention | Is there evidence that controlling for this co-intervention was unnecessary (e.g. because it was not administered)? | Is presence of this co-intervention likely to favour outcomes in the experimental intervention or the comparator |

| | | |
|---|---|---|
| None | - | Favour experimental / Favour comparator / No information |

Risk of bias assessment. Responses underlined in green are potential markers for low risk of bias, and responses in red are potential markers for a risk of bias. Where questions relate only to sign posts to other questions, no formatting is used.

| Signalling questions | Description | Response options |
|---|---|---|
| **Bias due to confounding** | | |
| 1.1 Is there potential for confounding of the effect of intervention in this study?<br>**If N/PN to 1.1:** the study can be considered to be at low risk of bias due to confounding and no further signalling questions need be considered | | Y / PY / PN / N |

| | | |
|---|---|---|
| **If Y/PY to 1.1**: determine whether there is a need to assess time-varying confounding: | Baseline confounding occurs when one or more pre-intervention prognostic factors predict the intervention received at start of follow up.<br>The study adopted "an inherent" process of randomly allocating patients to groups (intervention, usual care, control).<br>"First, consenting patients who happen to be allocated to the teams that have the intervention clinical pharmacists will be naturally considered<br>in the intervention arm. Second, consenting patients who happen to be allocated to the teams that have the non-intervention clinical pharmacists will be considered in the usual care arm. Finally, consenting patients who are discharged on weekends and after the clinical pharmacists' working hours will be naturally randomised into<br>the control arm". The possibility that allocation of patients to different teams according to their complexity (baseline characteristics) or that those discharged over the weekend may be less complex and don't need intervention can't be ruled out. Although "baseline confounding" in this context is more relevant to observational/non-experimental studies but it should be considered in this context. But time varying confounding, which occurs when follow-up time is split according to the intervention received is unlikely a concern in a prospective quasi-randomized study. | |
| 1.2. Was the analysis based on splitting participants' follow up time according to intervention received?<br>**If N/PN**, answer questions relating to baseline confounding (1.4 to 1.6)<br>**If Y/PY**, go to question 1.3. | **N/PN** | NA / Y / PY / PN / N / NI |

| | | |
|---|---|---|
| 1.3. Were intervention discontinuations or switches likely to be related to factors that are prognostic for the outcome?<br>**If N/PN**, answer questions relating to baseline confounding (1.4 to 1.6)<br>**If Y/PY**, answer questions relating to both baseline and time-varying confounding (1.7 and 1.8) | | NA / Y / PY / PN / N / NI |
| **Questions relating to baseline confounding only** | | |
| 1.4. Did the authors use an appropriate analysis method that controlled for all the important confounding domains? | Logistic regression analysis was used to adjust for baseline imbalances Healthcare utilization outcome was adjusted for age, gender, PCI, CAD, diabetes, obesity, type of ACS, hypertension, hyperlipidemia). Adherence was not adjusted for any confounder. | NA / Y / PY / PN / N / NI |
| 1.5. **If Y/PY to 1.4**: Were confounding domains that were controlled for measured validly and reliably by the variables available in this study? | | NA / Y / PY / PN / N / NI |
| 1.6. Did the authors control for any post-intervention variables that could have been affected by the intervention? | | NA / Y / PY / PN / N / NI |
| **Questions relating to baseline and time-varying confounding** | | |
| 1.7. Did the authors use an appropriate analysis method that controlled for all the important confounding domains and for time-varying confounding? | | NA / Y / PY / PN / N / NI |
| 1.8. **If Y/PY to 1.7**: Were confounding domains that were controlled for measured validly and reliably by the variables available in this study? | | NA / Y / PY / PN / N / NI |
| **Risk of bias judgement** | Serious | Low / Moderate / Serious / Critical / NI |

| | | |
|---|---|---|
| Optional: What is the predicted direction of bias due to confounding? | | Favours experimental / Favours comparator / Unpredictable |

| Bias in selection of participants into the study | | |
|---|---|---|
| 2.1. Was selection of participants into the study (or into the analysis) based on participant characteristics observed after the start of intervention?<br>**If N/PN to 2.1:** go to 2.4<br>2.2. **If Y/PY to 2.1**: Were the post-intervention variables that influenced selection likely to be associated with intervention?<br>2.3 **If Y/PY to 2.2**: Were the post-intervention variables that influenced selection likely to be influenced by the outcome or a cause of the outcome? | Selection of participants was done at baseline before allocation participants to groups. | Y / PY / PN / N / NI<br><br>NA / Y / PY / PN / N / NI<br><br>NA / Y / PY / PN / N / NI |
| 2.4. Do start of follow-up and start of intervention coincide for most participants? | | Y / PY / PN / N / NI |
| 2.5. **If Y/PY to 2.2 and 2.3, or N/PN to 2.4**: Were adjustment techniques used that are likely to correct for the presence of selection biases? | N/A | NA / Y / PY / PN / N / NI |
| **Risk of bias judgement** | Low | Low / Moderate / Serious / Critical / NI |

| | | |
|---|---|---|
| Optional: What is the predicted direction of bias due to selection of participants into the study? | | Favours experimental / Favours comparator / Towards null /Away from null / Unpredictable |

| **Bias in classification of interventions** | | |
|---|---|---|
| 3.1 Were intervention groups clearly defined? | Intervention, usual care, and control arms were reasonably described (interventions received in each group). Criteria for considering individuals to have received each intervention was clear. | Y / PY / PN / N / NI |
| 3.2 Was the information used to define intervention groups recorded at the start of the intervention? | Yes. They were recorded at protocol stage before the start of the intervention. | Y / PY / PN / N / NI |
| 3.3 Could classification of intervention status have been affected by knowledge of the outcome or risk of the outcome? | This is less relevant in a quasi-experimental study where participants were allocated to intervention before knowledge of the outcome | Y / PY / PN / N / NI |
| **Risk of bias judgement** | Low | Low / Moderate / Serious / Critical / NI |
| Optional: What is the predicted direction of bias due to classification of interventions? | | Favours experimental / Favours comparator / Towards null /Away from null / Unpredictable |

| Bias due to deviations from intended interventions | | |
|---|---|---|
| **If your aim for this study is to assess the effect of assignment to intervention, answer questions 4.1 and 4.2** | | |
| 4.1. Were there deviations from the intended intervention beyond what would be expected in usual practice? | | Y / PY / PN / N / NI |
| 4.2. **If Y/PY to 4.1**: Were these deviations from intended intervention unbalanced between groups *and* likely to have affected the outcome? | | NA / Y / PY / PN / N / NI |
| **If your aim for this study is to assess the effect of starting and adhering to intervention, answer questions 4.3 to 4.6** | | |
| 4.3. Were important co-interventions balanced across intervention groups? | | Y / PY / PN / N / NI |
| 4.4. Was the intervention implemented successfully for most participants? | | Y / PY / PN / N / NI |
| 4.5. Did study participants adhere to the assigned intervention regimen? | | Y / PY / PN / N / NI |
| 4.6. **If N/PN to 4.3, 4.4 or 4.5**: Was an appropriate analysis used to estimate the effect of starting and adhering to the intervention? | | NA / Y / PY / PN / N / NI |
| **Risk of bias judgement** | Low | Low / Moderate / Serious / Critical / NI |
| Optional: What is the predicted direction of bias due to deviations from the intended interventions? | | Favours experimental / Favours comparator / Towards null /Away from null / Unpredictable |

| Bias due to missing data |
|---|

| | | |
|---|---|---|
| 5.1 Were outcome data available for all, or nearly all, participants? | Outcomes reported for all enrolled subjects and "intention to treat" analysis. | Y / PY / PN / N / NI |
| 5.2 Were participants excluded due to missing data on intervention status? | | Y / PY / PN / N / NI |
| 5.3 Were participants excluded due to missing data on other variables needed for the analysis? | | Y / PY / PN / N / NI |
| 5.4 **If PN/N to 5.1, or Y/PY to 5.2 or 5.3**: Are the proportion of participants and reasons for missing data similar across interventions? | | NA / Y / PY / PN / N / NI |
| 5.5 **If PN/N to 5.1, or Y/PY to 5.2 or 5.3**: Is there evidence that results were robust to the presence of missing data? | | NA / Y / PY / PN / N / NI |
| **Risk of bias judgement** | Low | Low / Moderate / Serious / Critical / NI |
| Optional: What is the predicted direction of bias due to missing data? | | Favours experimental / Favours comparator / Towards null /Away from null / Unpredictable |

| **Bias in measurement of outcomes** | | |
|---|---|---|
| 6.1 Could the outcome measure have been influenced by knowledge of the intervention received? | Outcome assessors were blinded to the intervention received and reported adherence from pharmacy dispensing records | Y / PY / PN / N / NI |

| | | |
|---|---|---|
| 6.2 Were outcome assessors aware of the intervention received by study participants? | | Y / PY / PN / N / NI |
| 6.3 Were the methods of outcome assessment comparable across intervention groups? | | Y / PY / PN / N / NI |
| 6.4 Were any systematic errors in measurement of the outcome related to intervention received? | PDC does not necessarily reflect treatment actually taken by patients but same method was used in both groups | Y / PY / PN / N / NI |
| **Risk of bias judgement** | Low | Low / Moderate / Serious / Critical / NI |
| Optional: What is the predicted direction of bias due to measurement of outcomes? | | Favours experimental / Favours comparator / Towards null /Away from null / Unpredictable |

| **Bias in selection of the reported result** | | |
|---|---|---|
| Is the reported effect estimate likely to be selected, on the basis of the results, from...<br><br>7.1. ... multiple outcome *measurements* within the outcome domain? | Authors planned to check adherence by at 3,6 and 12 months using 3 different methods (PDC, SELF-REPORT, Administration of the Adherence to Refills and Medications Scale (ARMS) BUT REPORTED only PDC at 6 months | Y / PY / PN / N / NI |
| 7.2 ... multiple *analyses* of the intervention-outcome relationship? | The protocol stated that (Two sensitivity analyses will be performed one using multiple imputations by chained equations for the missing data and the other using per protocol methodology) but results not presented. | Y / PY / PN / N / NI |
| 7.3 ... different *subgroups*? | | Y / PY / PN / N / NI |

| | | |
|---|---|---|
| **Risk of bias judgement** | Serious | Low / Moderate / Serious / Critical / NI |
| Optional: What is the predicted direction of bias due to selection of the reported result? | | Favours experimental / Favours comparator / Towards null /Away from null / Unpredictable |

| **Overall bias** | | |
|---|---|---|
| **Risk of bias judgement** | **Serious** | Low / Moderate / Serious / Critical / NI |
| Optional: What is the overall predicted direction of bias for this outcome? | | Favours experimental / Favours comparator / Towards null /Away from null / Unpredictable |

This work is licensed under a Creative Commons Attribution-NonCommercial-NoDerivatives 4.0 International License.

**Table 1a: Description of the intervention (arranged by study design, type/components of the interventions and year of publication):**

| First author, publication year, country | Practice setting | Number of intervention components | Description of the intervention (I) and control (C) groups | Strategies to improve or maintain intervention fidelity | Extent of intervention fidelity |
|---|---|---|---|---|---|
| **Randomized Controlled trials (RCTs)** | | | | | |
| **Interventions offered during hospital admission** | | | | | |
| Zerafa N (Zerafa et al., 2011) 2011 Malta | Cardiac Surgical Ward and Outpatients Clinic Of an acute general teaching hospital | 1 | I: pharmacist **counseling upon discharge** (verbal+ medication photographs and discharge medication chart) in addition to the usual advice given to patients.<br><br>C: usual care (the doctor gave verbal and written advice to patients regarding medication regimen, the physiotherapy team explained exercise regimens and the rehabilitation nurse discussed the lifestyle changes expected post-surgery). | Not described | Not described |
| Al-Hashar A(Al-Hashar et al., 2018) 2018 Oman | Tertiary care academic hospital<br><br>(Medical wards) | 4 | I: pharmacists obtained medication history upon admission, (1) medication reconciliation on admission, (2) medication reconciliation on discharge, identification of unintentional discrepancies, judged after discussion with the prescriber, and efforts were made to reconcile those discrepancies, (3) provision of bedside medication delivery, (4) counselling and take-home medication list and addressed adherence concerns upon discharge.<br><br>C: standard care, **included ward-based pharmacist coverage** in the form of a general, simple (type 1) to moderate (type 2A) medication review (as defined by Pharmaceutical Care Network in Europe (PCNE). On discharge, patients were handed their discharge papers by nurses or doctors. Patients or their caregivers collected their medications at the pharmacy window and received basic instructions on dose and duration. | Not described<br><br>All steps in each arm were carried out by the same pharmacist for all patients. | The intervention was fully delivered to 243/286 patients<br><br>(Remaining 43 patients left without being provided with medication reconciliation, medication counselling, or list of medications on discharge because they were discharged on weekends or outside working hours) |
| Salameh L (Salameh et al., 2019) 2019 Jordan | Tertiary care teaching hospital<br><br>(Internal medicine Departments) | 2 | I: (1) admission medication reconciliation (pharmacists identified medication discrepancies and provided recommendations to the responsible clinicians to resolve them using a structured written consult form), (2) education about medications at discharge.<br><br>C: not described. Only mentioned “no intervention was Provided (to resolve identified medication discrepancies)” | Data were collected by a well-trained pharmacists (one of the authors) who received an extensive training about how to conduct a reconciliation service. | Not described<br><br>(Note: responsible clinicians accepted 92.8% and implemented only 54.7% of submitted pharmacists’ recommendations) |

| | | | | | |
|---|---|---|---|---|---|
| Abu Hammour K (Abu Hammour et al., 2022) 2022 Jordan | Tertiary care teaching hospital<br><br>(Surgical department) | 1 | I: (1) admission medication reconciliation (pharmacists identified medication discrepancies and provided recommendations to the responsible clinicians to resolve them using a consult form.<br><br>C: not described. Only described "pharmacists identified medication discrepancies during admission medication reconciliation, but no recommendation written by pharmacists to solve these discrepancies unless classified as severe (class 3). No class 3 discrepancies were identified so no clinical pharmacists' interventions were performed. | Data were collected by 2 clinical pharmacist preceptors.<br>trained in data collection and in identifying and resolving medication discrepancies in a standardized, systematic manner.<br><br>Training included a didactic lecture, followed by a simulation training session.<br><br>To ensure consistency in identifying medication discrepancies, some cases from each of the clinical pharmacists were re-evaluated independently by another researcher; no differences were found. | Not described.<br><br>(Note: 38% of the submitted recommendations, were accepted by the treating physician, and all of them led to resolution of the medication discrepancy) |
| **Interventions commenced during hospital admission that include continuing support post-discharge** | | | | | |
| Ibrahim M (Ibrahim, 2012) 2012 Egypt | Teaching hospital<br><br>(General medicine service) | 3 | I: (1) discharge medication regimens were compared with preadmission regimens and all discrepancies were reconciled with the medical team's help. Patients were screened for previous DRPs, including non-adherence, lack of efficacy, and side effects, (2) pharmacist reviewed<br>the indications, directions for use, and potential adverse effects of each discharge medication with the patient, (3) telephone follow-up 3-4 days after discharge during which the clinical pharmacist asked about medication adherence, possible ADEs, and adherence with scheduled follow-up visits and laboratory appointments.<br><br>C: routine review of medication orders **by a ward-based pharmacist** at the time of discharge. Discharge counseling typically focused on directions to use medications and may have included a discussion of indications or potential side effects, especially for new medications. | Not described | Not described |
| Sanii Y (Sanii et al., 2016) 2015 Islamic Republic of Iran (IRI) | Teaching hospital<br><br>(Respiratory ward) | 3 | I: (1) discharge patients' education on health<br>condition and drug therapy with provision of written asthma education materials (2) comparison of discharge medication with preadmission regimens and reconciliation of discrepancies with the medical team's help, (3) phone call follow-up two weeks post discharge to determine if patient follows discharge medication's instruction of use.<br><br>C: usual care, discharged without any intervention, and they had no access to the pharmacist | Not described | Not described.<br>(Note: 90 out of 100 randomized to intervention group received the allocated intervention, remaining 10 were discharged without prescription, and 91 out of 100 in the control group) |

| | | | | | |
|---|---|---|---|---|---|
| Salmany S (Salmany et al., 2018) 2017 Jordan | Teaching cancer center | 2 | I: (1) a telephone follow-up call by pharmacist. Pharmacist communicated patient-reported medication related adverse effects to the treating physician and discussed potential interventions and then contacted the patient to deliver the new treatment plan.<br><br>C: No telephone follow-up call by pharmacist.<br><br>** (2) **all patients received extensive counseling by a specialized clinical pharmacist** prior to discharge. (Both groups) as part of standard of care. | The content of the call followed a pre-determined set of questions. | The intervention (phone follow-up) was delivered to 90% of patients in intervention group.<br><br>mean time (SD) of the interventional telephone call was 3 (1.7) minutes.<br><br>Time taken to make calls with the treating physicians to discuss medication-related adverse effects that emerged after discharge and time taken to call the patient again and deliver new treatment plans was not recorded. |
| Karaoui L (Karaoui et al., 2021) 2021 Lebanon | Tertiary care teaching hospital | 3 | I: (1) pharmacist-driven discharge counseling with a focus on anticoagulant medication (verbal and written education (two educational pamphlets, 1st was general about all anticoagulants and 2nd was specific to prescribed anticoagulant)) +standard of care, (2) review of patient discharge medications, (3) telephone call follow-up on day 3 post-discharge.<br><br>C: standard of care discharge counseling (nurse-driven) on anticoagulants at the hospital (handing the patients their discharge prescription along with discharge instructions, no written educational material handed to patients as part of the standard of care) | All pharmacists underwent training before they started counseling patients to ensure standardization of the information delivered during the counseling session. Two educational pamphlets used for education.<br><br>Used post-discharge telephone call follow-up script for anticoagulation education adapted from the Agency for Healthcare Research and Quality (AHRQ) was used.<br><br>Monitored documented elements of patient education in the medical record. | The intervention (phone follow-up) was delivered to 92% of patients in intervention group.<br><br>Monitoring elements of counseling session (intervention group: only 32% educated about anticoagulation reversal agents and 31%<br>informed about next appointment date with physician) |
| Marzoog H (Marzoog et al., 2021) 2021 Iraq | Tertiary care teaching hospital | 2 | I: pharmacist- based program for assessment and review starting from 30 minutes pre-hospital discharge, then during subsequent post hospital discharge visits 1 (2 weeks), visits 2 (4 weeks), visits 3 (8weeks) and visits 4 (12weeks).<br>The following details were obtained by direct communication with the authors (first and second authors): (1) At discharge education about disease and drug therapy for patients, and their caregivers, (2) After discharge education and detection, prevention, and solving any drug related problems whether actual or potential via direct communication with the caregiver and physician.<br>C: usual medical care and follow up achieved by the medical staff only without pharmacist attachment. | Not described | Not described |
| Bawazeer G (Bawazeer et al., 2021) | Tertiary care teaching hospital | 3 | I: student-led (advanced pharmacy practice experience (APPE) program interns in their last professional year). Intervention consisted of (1) medication reconciliation at discharge, (2) a patient interview | All students were required to complete reading assignments and attend a half-day training workshop to ensure a sufficient | 74% of patients in the standard of care group received usual care discharge education, while 61% of |

| | | | | | |
|---|---|---|---|---|---|
| 2021<br>Kingdom of Saudi Arabia (KSA) | | | and counselling (provision of printed educational materials about insulin and/or warfarin and an updated list of the patient's medications), (3) phone call follow-up at 72h post discharge.<br><br>C: received traditional education. At a minimum, patients with diabetes were often seen by a health educator and/or a dietitian to receive advice on their insulin regimen and/or diet. **Some units provided clinical pharmacists discharge counselling;** however, staff workload and time constraints did not allow the delivery of discharge counselling to every patient. | competency level. The workshop covered interviewing techniques,<br>insulin types and administration technique, and medication specific educational material. Student readiness was assessed against a competency checklist and an objective assessment exam with a required passing score of 100%. Students used an iPad preloaded with visual PowerPoint presentation on insulin and warfarin to ensure consistent delivery of critical counselling information for all patients and across participating students. All phone calls were scripted and approved by the research team to ensure uniformity of questions asked and data collected.<br><br>Described fidelity elements such as dose (duration) | patients in the intervention group received such education.<br><br>Intervention group: mean (SD time spent on discharge counselling sessions was 5.3 (3.8) minutes.<br><br>Mean (SD) time to the 72-hr follow-up calls in the intervention group was 4.9 (2.4) days, and mean (SD) time spent on these calls was 5 (1.0) minutes. |
| **Quasi-experimental studies** | | | | | |
| **Interventions commenced during hospital admission that include continuing support post-discharge** | | | | | |
| El Hajj M<br>(El Hajj et al., 2023)<br>2023<br>Qatar | Tertiary care hospital<br><br>(Heart hospital) | 4 | I: clinical pharmacist-delivered structured intervention at discharge consisting of: (1) medication reconciliation, (2) detected and resolved drug therapy problems (DTP), (3) structured counselling (provided a personalized medication timetable, a pill box and information leaflets), (4) two phone follow-up sessions (30-min each) at 4 weeks and 8 weeks after hospital discharge.<br><br>Usual care: clinical pharmacist-delivered usual care at discharge, which included: medication reconciliation, identification and resolution of DTPs and discharge counseling.<br><br>C: regular discharge education by nurses and/or treating physicians (included patients discharged during weekends and/or at times outside the clinical pharmacists' working hours.) | The study team provided the intervention pharmacists.<br>with a 3-hour orientation session (covered a refresher on the following: the current guidelines on the secondary prevention of ACS, non-pharmacological management, pharmaceutical care's basic concepts, communication and counselling skills, medication reconciliation, medication adherence assessment and strategies to improve medication adherence, the study protocol and the study forms that the pharmacists would need to fill, pharmacists were orientated to provide the intervention in a standardized fashion, while tailoring it to the patient's needs), pilot testing on 2 patients with audio recording of counseling sessions and individualized feedback to intervention pharmacists. | Not described |
| **Interventions commenced post-discharge** | | | | | |

| | | | | | |
|---|---|---|---|---|---|
| Al-Mahroos M (Al-Mahroos et al., 2017) 2017 Iraq | Outpatient clinic | 1 | I: patient instructions and education, INR monitoring, home medicine review to identify and resolve any post discharge medications related issues and communication with prescriber physician for warfarin dose adjustment if any.<br><br>C: usual medical care and follow up achieved by patient routine visit to a physician working in the center for INR monitoring and dose adjustment. | Not described | Not described |

Adapted from Coxeter, Peter et al. (Coxeter et al., 2015)

## Detailed description of the results:

### Healthcare utilization:

We planned initially to report 30 days hospital readmission, however due to variations of the

outcomes identified, and the limited data available for the pre-specified outcome, we reported healthcare utilization (including hospital readmission and/or ED visits) as defined in individual studies.

Healthcare utilization was reported in 8 RCTs and 1 quasi-experimental study.

It was assessed at 30-days post discharge in 7 studies, and at 12 weeks and 6 months in the remaining two studies. (table 1)

**Evidence from RCTs:**

**Description of the outcome according to the type of interventions:**

**Interventions offered during hospital admission:**

Two RCTs (Al-Hashar et al., 2018; Salameh et al., 2019) that implemented interventions targeting admission and discharge among patients admitted to medical wards evaluated healthcare utilization at 30-days post-discharge. The trial by Al-Hashar et al (Al-Hashar et al., 2018), implemented 4 interventions and reported the number (median (IQR)) of emergency department (ED) visits, unplanned hospital visits, unplanned hospital readmissions, and a composite of the number of ED visits, hospital readmissions, and unplanned hospital visits. None of which differed significantly between the intervention and control arms. We contacted the corresponding author to inquire about the actual number of readmitted patients but did not receive any response. Nonetheless, the trial found a significant reduction in hospitalization due to preventable ADE (I: 6 (2.1%) vs. C: 16 (5.3%), P= 0.040).

The second trial (Salameh et al., 2019), which implemented 2 interventions, reported comparable proportions of hospital readmissions and ED visits between the intervention and control groups.

**Interventions commenced during hospital admission that include continuing support post-discharge**

**-Studies implementing 3 interventions**:

(Discharge reconciliation or review, education, and post discharge phone follow-up):

In the study by Bawazeer et al, (Bawazeer et al., 2021) the intervention was delivered by undergraduate pharmacy students, under the supervision of two clinical pharmacists. The trial targeted patients discharged on insulin, warfarin or both. Hospital readmission within 30-days post-discharge was comparable between the intervention and control groups (15% vs. 23%, respectively, P = 0.48).

Ibrahim et al, (Ibrahim, 2012) enrolled patients from general medicine service and reported a composite outcome of 30-days ED visit or readmission, which was comparable between the intervention and control groups (24% vs. 28%). A similar finding was reported for medication-related ED visits or readmissions (intervention: 6% vs. control 8%). However, preventable medication-related ED visits or hospital readmissions were significantly reduced in the intervention group. (I: 2% vs. C: 9%, P= 0.03).

Sanii et al, (Sanii et al., 2016) targeted patients discharged from respiratory ward on certain medications (inhalers, antihypertensive medications, digoxin or antiplatelets) and reported the number of patients experiencing the composite outcome of medication-related ED visits or hospital readmission, which was 0 in the intervention group and 8 in the control group. However, the statistical testing of the difference was not reported.

Karaoui et al, (Karaoui et al., 2021) enrolled patients discharged on oral anticoagulants for therapeutic indications and evaluated all-cause readmissions, including unplanned physician's clinic visits. The difference between the intervention and control groups was insignificant (15% vs. 12%, respectively, Odds ratio (OR) 0.847 (0.380–1.886), P=0.802). The study also reported comparable anticoagulant—related readmissions between the two groups.

**-Studies implementing 2 interventions:**

(Discharge counseling and post-discharged follow-up):

Marzoog et al, (Marzoog et al., 2021) evaluated pharmacist delivered education upon discharge, followed by four follow-up clinic visits over 12 weeks, and reported readmission due to acute heart failure exacerbation in 24% of the intervention group versus 60% in the control group. The statistical testing of the difference was not reported.

Salmany et al, (Salmany et al., 2018) included oncology patients and demonstrated comparable proportions of ED visits and hospital readmissions between the intervention and control groups.

**Evidence from quasi-experimental studies:**

Healthcare utilization was assessed as the primary outcome in the study by El Hajj et al, (El Hajj et al., 2023) which evaluated 3 arms (intervention, usual care and control arms). The study did not find a significant difference in the odds of all-cause hospitalizations at 6 months neither between the intervention and usual care groups (adjusted odds ratio (aOR) 1.701, 95% CI 0.888- 3.257, P= 0.109) nor between the intervention and control groups (aOR 1.744, 95% CI 0.876-3.474, P= 0.114). However, patients in the control group experienced a significantly higher odds of cardiac related hospitalization at 6 months (OR 2.428:95% CI 1.116–5.282, p = 0.025) compared to the intervention group. A similar finding was reported when the intervention group was compared to the control and usual care groups combined (OR 2.140; 95% CI 1.062–4.312 p = 0.033).

For the meta-analysis, the three studies that reported the proportion of patients experiencing all cause 30-days readmission were pooled. The Odds ratio was 0.71 (95% CI 0.48-1.06), P=0.09. A finding that was not statistically significant.

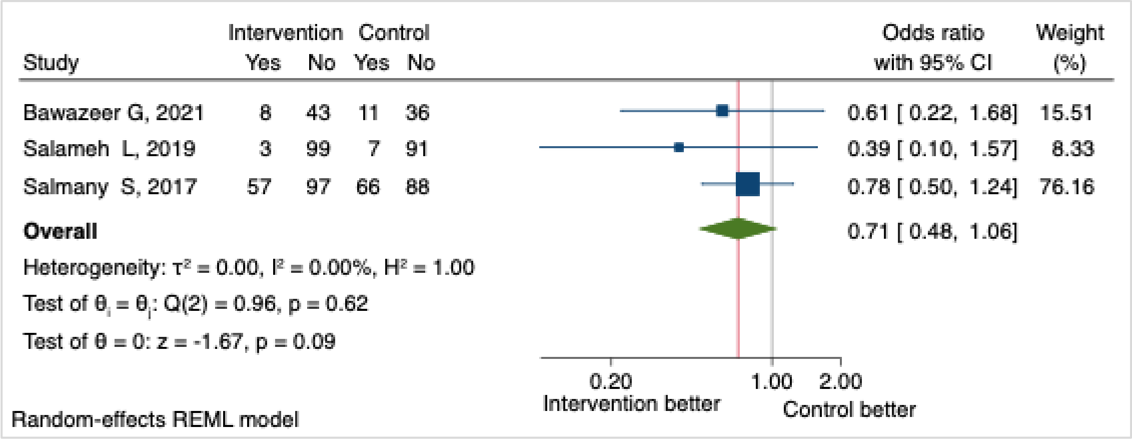


**Figure 4:** Proportion of patients with all-cause hospital readmission at 30-days.

## Adherence:

The impact of pharmacists' intervention at TOC on patients' adherence to medications post discharge was evaluated by 3 RCT and 2 quasi-experimental studies. It was the primary outcome of 2 RCTs (Sanii et al., 2016; Zerafa et al., 2011).

Only one study provided medication adherence aid to participants in the intervention group(El Hajj et al., 2023)

**Interventions commenced during hospital admission:**

Zerafa et al, (Zerafa et al., 2011) evaluated the impact of discharge counseling delivered by undergraduate pharmacy students on adherence 8 weeks post-hospital discharge.

Adherence was assessed by "Assessing Patient Compliance Questionnaire", a researcher developed questionnaire. Compliance was calculated by adding up the correct answers (dose, dosage interval and instructions) of each medication prescribed. Results were presented as percentage of the total number of correct and incorrect answers. The mean percentage compliance in the intervention and the control groups was 88.2% (95%CI 83.3– 93.2) and 66.4% (95%CI 59.0-73.9), respectively, P<0.001. The authors also reported the percentages of patients who never missed medication doses (control: 67.5% vs. intervention: 87.5%, P=0.032), who reported taking medicines as prescribed (control: 30.0% vs. Intervention: 50.0%, P=0.009) and who never stopped medications abruptly (control: 77.5% vs. control 90.0%, P=0.146). However, the authors did not report how these findings were assessed and whether they were part of the questionnaire used.

**Interventions commenced during hospital admission that include continuing support post-discharge**

Two RCTs that employed three pharmacist interventions (discharge reconciliation, education and post-discharge phone follow up). Adherence was assessed 30-days post discharge.

Ibrahim et al, (Ibrahim, 2012) assessed adherence by asking the patients, who were aware of the intervention assignment, if they received the medications as prescribed the day prior to the follow-up call and reported a significant difference in the number (%) of patients who were non-adherent to at least one medication between the intervention and control groups (16 (13%) vs. C: 30 (24%), respectively, P value= 0.026).

The trial by sanii et al, (Sanii et al., 2016) assessed adherence to inhaler medications using the medication adherence rating scales (MARS) questionnaire (10 questions) and reported a significant difference in the mean (SD) adherence score (Intervention: 93.2 (9.2) vs. Control: 50.3 (27.1), P=0.010. However, the version of the MARS questionnaire used was not specified, and the reported result did not match that of the Medication adherence report scale for asthma (MARS-A), which defines high adherence as a score of 4.5 or higher (R Horne & Hankins, 2004), which limits the interpretation of the current findings. Moreover, it is unclear if the questionnaire was validated in Persian language.

**Evidence from quasi-experimental studies:**

In the study by Al-Hajj et al, (El Hajj et al., 2023) the impact of structured discharge education and post-discharge follow-up on adherence at 6 months post discharge was evaluated by the proportion of days covered (PDC). A patient was considered adherent to all ACS secondary prevention medications (aspirin, β-blocker, statin, ACEI or ARB if applicable) if PDC was greater than 75%. The study did not find a significant difference between the three evaluated arms: intervention (60.7%), usual care (60.0%), control (50.0%), P= 0.156.

Al-Mahroos. et al, evaluated adherence to warfarin among patients followed by a pharmacist-lead anticoagulation clinic compared to usual care, and reported a 100% adherence in the intervention group over 90-days compared to 87% in the comparison group, P= 0.002.

However, the method of assessing adherence was not reported. Upon contacting the study authors, it was revealed that adherence was assessed by self-report of the number of doses missed in the previous month.

**References:**


Abu Hammour, K., Abu Farha, R., Ya'acoub, R., Salman, Z., & Basheti, I. (2022). Impact of Pharmacist-Directed Medication Reconciliation in Reducing Medication Discrepancies: A Randomized Controlled Trial. *Can J Hosp Pharm*, *75*(3), 169-177. https://doi.org/10.4212/cjhp.3143

Al-Hashar, A., Al-Zakwani, I., Eriksson, T., Sarakbi, A., Al-Zadjali, B., Al Mubaihsi, S., & Al Za'abi, M. (2018). Impact of medication reconciliation and review and counselling, on adverse drug events and healthcare resource use. *Int J Clin Pharm*, *40*(5), 1154-1164. https://doi.org/10.1007/s11096-018-0650-8

Al-Mahroos, M. I., Abdulridha, M. K., Alhaleem, M. R., Al-Mahroos, M., Abdulridha, M., & Alhaleem, M. (2017). Evalution of a designed community-based postdischarge warfarin management protocol on Iraqi patients. *International Journal of Pharmaceutical Sciences and Research*, *8*(5), 2323-2332.

Bawazeer, G., Sales, I., Alsunaidi, A., Aljahili, S., Aljawadi, M. H., Almalag, H. M., Alkofide, H., Adam Mahmoud, M., Alayoubi, F., & Aljohani, M. (2021). Student-Led discharge counseling program for High-Risk medications in a teaching hospital in Saudi Arabia: A pilot study. *Saudi Pharm J*, *29*(10), 1129-1136. https://doi.org/10.1016/j.jsps.2021.08.004

Coleman, E. A., Smith, J. D., Frank, J. C., Eilertsen, T. B., Thiare, J. N., & Kramer, A. M. (2002). Development and testing of a measure designed to assess the quality of care transitions. *Int J Integr Care*, *2*, e02. https://doi.org/10.5334/ijic.60

Coxeter, P., Del Mar, C. B., McGregor, L., Beller, E. M., & Hoffmann, T. C. (2015). Interventions to facilitate shared decision making to address antibiotic use for acute respiratory infections in primary care. *Cochrane Database of Systematic Reviews*(11).

El Hajj, M. S., Kaddoura, R., Abu Yousef, S. E. A., Orabi, B., Awaisu, A., AlYafei, S., Shami, R., & Mahfoud, Z. R. (2023). Effectiveness of a structured pharmacist-delivered intervention for patients post-acute coronary syndromes on all-cause hospitalizations and cardiac-related hospital readmissions: a prospective quasi-experimental study [Article in Press]. *Int J Clin Pharm*. https://doi.org/10.1007/s11096-023-01538-4

Horne, R. (2006). Compliance, adherence, and concordance: implications for asthma treatment. *Chest*, *130*(1 Suppl), 65S-72S. https://doi.org/10.1378/chest.130.1_suppl.65S

Horne, R., & Hankins, M. (2004). The medication adherence report scale. *University of Brighton: Brighton, UK*.

Ibrahim, O. H. M. (2012). Impact of Clinical Pharmacist Intervention on Decreasing Incidence of Preventable Adverse Drug Events after Hospital Discharge. *Advances in Pharmacoepidemiology & Drug Safety*, *01*(02). https://doi.org/10.4172/2167-1052.1000111

Karaoui, L. R., Ramia, E., Mansour, H., Haddad, N., & Chamoun, N. (2021). Impact of pharmacist-conducted anticoagulation patient education and telephone follow-up on transitions of care: a randomized controlled trial. *BMC Health Serv Res*, *21*(1), 151, Article 151. https://doi.org/10.1186/s12913-021-06156-2

Leape, L. L., Lawthers, A. G., Brennan, T. A., & Johnson, W. G. (1993). Preventing medical injury. *QRB Qual Rev Bull*, *19*(5), 144-149. https://doi.org/10.1016/s0097-5990(16)30608-x

Marzoog, H. F., Abdulridha, M. K., & Nassir, S. F. (2021). Improvement of hospital discharge summary among patients with moderate to severe acute heart failure: Pharmacist –based intervention [Article]. *International Journal of Pharmaceutical Research*, *13*(02), 32-42. https://doi.org/10.31838/ijpr/2021.13.02.012

National Coordinating Council for Medication Error Reporting and Prevention. (2023). About Medication Errors. https://www.nccmerp.org/about-medication-errors

Orbell, S., Schneider, H., Esbitt, S., Gonzalez, J. S., Gonzalez, J. S., Shreck, E., Batchelder, A., Gidron, Y., Pressman, S. D., Hooker, E. D., Wiebe, D. J., Rinehart, D., Hayman, L. L., Meneghini, L., Kikuchi, H., Kikuchi, H., Desouky, T. F., McAndrew, L. M., Mora, P. A., Bruce, B., Luger, T. M., Allebeck, P., Allebeck, P., Allebeck, P., Carrasquillo, O., McAlister, A. L., Molina, K. M., Birch, S., Gafni, A., Baumann, L. C., Karel, A., Sollins, H., Hjortsberg, C., Sanders, L., Gidron, Y., Marcus, E. N., Carrasquillo, O., Tran, V., Allebeck, P., Wit, M., Hajos, T., Gidron, Y., Rarback, S., Wallhagen, M., Wallhagen, M., Ye, S., Newman, J., Whang, W., Hamer, M., Smith, T. W., DeBerard, S., Shapiro, P. A., Chida, Y., Sabol, V., Ginty, A. T., Thayer, J. F., Kanda, Y., Newman, J., Brintz, C., Whittaker, T., Wessel, J., Rodriguez-Murillo, L., Salem, R. M., Matsuyama, Y., Turner, J. R., Schneiderman, N., Ruiz, J., Garza, M., Smith, L., DeBerard, S., Overstreet, N., Mitchell, J. W., Carrasquillo, O., Rodriguez, O., Gaab, J., Ye, S., Rodriguez-Murillo, L., Salem, R. M., Gidron, Y., Wolf, O. T., Croom, A., Pellosmaa, H. B., Desouky, T. F., Palmer, S. C., Henderson, K. M., Everson-Rose, S. A., Clark, C. J., Everson-Rose, S. A., Clark, C. J., Henderson, K. M., Everson-Rose, S. A., Clark, C. J., Henderson, K. M., Henderson, K. M., Everson-Rose, S. A., Clark, C. J., Bakdash, J. Z., Drews, F. A., Salem, R. M., Rodriguez-Murillo, L., García, L. I., Roane, L., Coons, M. J., Parker, A., Flannery, K., Carroll, D., Rosenberg, L., Clark, M. S., Fortenberry, K. T., Jansen, K. L., Parker, A., Heaney, J., Beaton, E. A., & Turner, J. R. (2013). Health Care Utilization. In M. D. Gellman & J. R. Turner

(Eds.), *Encyclopedia of Behavioral Medicine* (pp. 909-910). Springer New York. https://doi.org/10.1007/978-1-4419-1005-9_885

Salameh, L. K., Abu Farha, R. K., Abu Hammour, K. M., & Basheti, I. A. (2019). Impact of pharmacist's directed medication reconciliation on reducing medication discrepancies during transition of care in hospital setting [Article]. *Journal of Pharmaceutical Health Services Research*, *10*(1), 149-156. https://doi.org/10.1111/jphs.12261

Salmany, S. S., Ratrout, L., Amireh, A., Agha, R., Nassar, N., Mahmoud, N., Rimawi, D., & Nazer, L. (2018). The impact of pharmacist telephone calls after discharge on satisfaction of oncology patients: A randomized controlled study. *J Oncol Pharm Pract*, *24*(5), 359-364. https://doi.org/10.1177/1078155217709616

Sanii, Y., Torkamandi, H., Gholami, K., Hadavand, N., & Javadi, M. (2016). Role of pharmacist counseling in pharmacotherapy quality improvement. *J Res Pharm Pract*, *5*(2), 132-137. https://doi.org/10.4103/2279-042X.179580

Zerafa, N., Zarb Adami, M., & Galea, J. (2011). Impact of drugs counselling by an undergraduate pharmacist on cardiac surgical patient's compliance to medicines. *Pharm Pract (Granada)*, *9*(3), 156-161. https://www.ncbi.nlm.nih.gov/pubmed/24367470
